# A sulfonimide derivative of bezafibrate as a dual inhibitor of cyclooxygenase-2 and PPARα

**DOI:** 10.3389/fphar.2024.1488722

**Published:** 2024-11-26

**Authors:** Alessandra Ammazzalorso, Stefania Tacconelli, Annalisa Contursi, Ulrika Hofling, Carmen Cerchia, Sara Di Berardino, Alessandra De Michele, Rosa Amoroso, Antonio Lavecchia, Paola Patrignani

**Affiliations:** ^1^ Department of Pharmacy, “G. d’Annunzio” University, Chieti, Italy; ^2^ Systems Pharmacology and Translational Therapeutics Laboratory, at the Center for Advanced Studies and Technology (CAST), and Department of Neuroscience, Imaging and Clinical Science, “G. d’Annunzio” University, Chieti, Italy; ^3^ Department of Pharmaceutical and Toxicological Chemistry, University of Naples “Federico II”, Naples, Italy

**Keywords:** COX-2, PPARα, whole blood, NSAIDs, coxibs, lipidomics of eicosanoids, colorectal cancer

## Abstract

**Background:**

PPARα and cyclooxygenase (COX)-2 are overexpressed in certain types of cancer. Thus, developing a dual inhibitor that targets both could be more effective as an anticancer agent than single inhibitors. We have previously shown that an analog of the bezafibrate named AA520 is a PPARα antagonist. Herein, we report the identification of AA520 as a potent COX-2 inhibitor using *in silico* approaches. In addition, we performed a thorough pharmacological characterization of AA520 towards COX-1 and COX-2 in different *in vitro* models.

**Methods:**

AA520 was characterized for inhibiting platelet COX-1 and monocyte COX-2 activity in human whole blood (HWB) and for effects on lipidomics of eicosanoids using LC-MS/MS. The kinetics of the interaction of AA520 with COX-2 was assessed in the human colon cancer cell line, HCA-7, expressing only COX-2, by testing the COX-2 activity after extensive washing of the cells. The impact of AA520 on cancer cell viability, metabolic activity, and cytotoxicity was tested using the MTT reagent.

**Results:**

In HWB, AA520 inhibited in a concentration-dependent fashion LPS-stimulated leukocyte prostaglandin (PG) E_2_ generation with an IC_50_ of 0.10 (95% CI: 0.05–0.263) μM while platelet COX-1 was not affected up to 300 μM. AA520 did not affect LPS-induced monocyte COX-2 expression, and other eicosanoids generated by enzymatic and nonenzymatic pathways. AA520 inhibited COX-2-dependent PGE_2_ generation in the colon cancer cell line HCA7. Comparison of the inhibition of COX-2 and its reversibility by AA520, indomethacin (a time-dependent inhibitor), acetylsalicylic acid (ASA) (an irreversible inhibitor), and ibuprofen (a reversible inhibitor) showed that the compound is acting by forming a tightly bound COX-2 interaction. This was confirmed by docking and molecular dynamics studies. Moreover, AA520 (1 μM) significantly reduced MTT in HCA7 cells.

**Conclusion:**

We have identified a highly selective COX-2 inhibitor with a unique scaffold. This inhibitor retains PPARα antagonism at the same concentration range. It has the potential to be effective in treating certain types of cancer, such as hepatocellular carcinoma (HCC) and renal cell carcinoma (RCC), where COX-2 and PPARα are overexpressed.

## 1 Introduction

There is strong evidence indicating that inflammation plays a crucial role in both the early stages of cancer development as well as in its progression toward metastasis (Patrignani and Patrono, 2015; Wang and Dubois, 2010a; 2010b). The activity of cyclooxygenase (COX)-2 contributes to inflammation by converting arachidonic acid (AA) into prostanoids, a family of lipid mediators. Among them, prostaglandin E_2_(PGE_2_) promotes tumorigenesis and metastasis via different mechanisms, and its inhibition causes anti-tumor effects by preventing invasion, proliferation, and angiogenesis and inducing apoptosis. The different biological responses of PGE_2_ are mediated by G protein-coupled receptors (EP1-4), expressed in a tissue-specific manner (Wang and Dubois, 2010a; 2010b; Santiso et al., 2024). In particular, EP2 and EP4 subtypes are involved in tumorigenesis, and currently, they represent interesting targets in the development of anticancer drugs (Santiso et al., 2024). Selective COX-2 inhibitors (collectively named coxibs) effectively reduce inflammation and cause anti-tumor effects through PGE_2_ biosynthesis inhibition. TPST 1495, a selective dual antagonist of EP2 and EP4, is in clinical development to improve the efficacy of immune checkpoint inhibitors in patients with advanced solid tumors (https://clinicaltrials.gov/study/NCT04344795).

Another important pathway in tumorigenesis is represented by the PPAR (Peroxisome Proliferator-Activated Receptor) family. PPARs are nuclear hormone receptors, including PPARα, PPARδ, and PPARγ, which are important in regulating cancer cell proliferation, survival, apoptosis, and tumor growth (Hong et al., 2019; Kaipainen et al., 2007; Spaner et al., 2013; Messmer et al., 2015). The PPARα subtype represents an interesting anticancer target due to its critical roles in metabolic regulation and immune function. PPARα is involved in several types of cancer through the activation of NF-kB and the regulation of fatty acid oxidation. PPARα promotes tumor cell growth and inhibits anticancer immunity (Tan et al., 2021). TPST-1120 is a first-in-class, oral, small molecule, competitive antagonist of PPARα, with nanomolar potency (IC_50_ 0.04 μM) for human PPARα and high specificity (>250-fold) for PPARα over the other PPAR isoforms (PPAR β/δ and γ). It has been shown to inhibit tumor growth in xenograft and syngeneic tumor models and to improve the efficacy of anti-PD-1 therapy in tumor reduction and durable antitumor immunity (Stock et al., 2017; Yarchoan et al., 2024). Recent First-in-human Phase I Trial results in patients with advanced tumors support its promising anticancer profile (Yarchoan et al., 2024).

**FIGURE 1:**
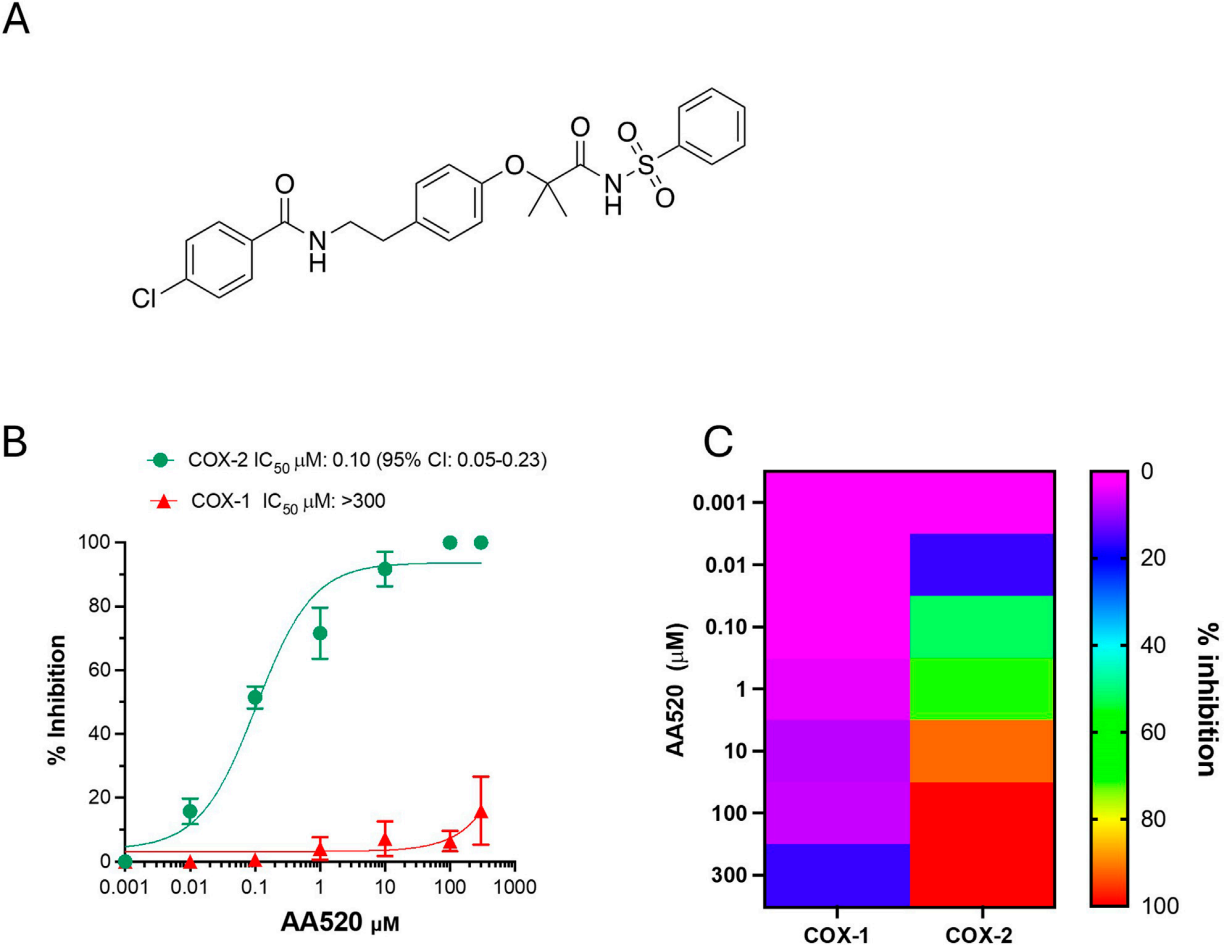
Effect of AA520 on the activity of COX-1 and COX-2 in human whole blood. **(A)** Chemical structure of AA520; **(B)** Concentration-response curves of inhibition in human whole blood of platelet COX-1 and LPS-induced monocyte COX-2 activity by AA520. Increasing concentrations of compound AA520 (0.001–300 µM) were incubated with heparinized whole blood samples, withdrawn from 4 healthy volunteers (2 females and 2 males) after suppressing the contribution of platelet COX-1 by adding aspirin (50 µM) *in vitro* solubilized in methanol and then evaporated, in the presence of LPS (10 μg/mL) for 24 h; after centrifugation, PGE_2_ levels were analyzed as an index of LPS-induced-COX-2 activity, by a specific RIA. Furthermore, AA520 (0.01–300 µM) was incubated with human whole blood (from the same individuals) and allowed to clot for 1 h at 37°C; after centrifugation, TXB_2_ levels were measured as an index of platelet COX-1 activity by a specific immunoassay. Results are depicted as percent inhibition (mean ± SEM, n = 3–4); **(C)** Heat map of % inhibition of COX-1 and COX-2 activities (mean values) versus increasing AA520 concentrations.

In hepatocellular carcinoma (HCC) and renal cell carcinoma (RCC), both COX-2 and PPARα are overexpressed (Chen et al., 2004; Cervello and Montalto, 2006; Abu Aboud et al., 2013). Thus, we have hypothesized that a molecule that inhibits both pathways could lead to improved anticancer effects. We have previously synthesized an analog of the bezafibrate named AA520 (Figure 1A) as a potent PPARα antagonist (Ammazzalorso et al., 2016). This compound was obtained by modifying the carboxyl portion of the bezafibrate and introducing a sulfonimide moiety. Using an *in silico* approach, we have found that AA520 binds COX-2. Here, we performed a thorough pharmacological characterization of AA520 towards COX-1 and COX-2 in different *in vitro* models. AA520 was also characterized for the capacity to interfere with other enzymatic and nonenzymatic pathways of AA by performing targeted lipidomics of eicosanoids by chiral liquid chromatography-mass spectrometry (LC-MS/MS) (Mazaleuskaya et al., 2018; Tacconelli et al., 2020a). The reversibility of the inhibition of COX-2 activity by AA520 was evaluated in the human colon cancer cell line, HCA-7, expressing only COX-2. Moreover, docking and molecular dynamics studies were performed. The impact of AA520 on cancer cell viability, metabolic activity, and cytotoxicity was tested using the MTT viability reagent.

## 2 Materials and methods

### 2.1 Materials

Acetonitrile (ACN), water (LC-MS grade), formic acid (FA), *n*-hexane, methanol, acetic acid, and isopropanol were from Carlo Erba Reagents, Milan, Italy. Standards of TXB_2_, PGE_2_, hydroxyeicosatetraenoic acid (HETE) s, leukotriene (LT) B_4_, their deuterated forms, 15R-lipoxin (LX) A_4_, and the immunoassay kit for the assessment of TXB_2_ (#501020) were from Cayman Chemical (Ann Arbor, Michigan, United States). ECL Western blotting Detection Reagents were from GE Healthcare (Milan, Italy). Dimethyl sulfoxide (DMSO), ethanol (EtOH), bovine serum albumin (BSA), NaCl, Triton X-100, Phenylmethylsulfonyl Fuoride (PMSF), Dulbecco’s Modified Eagle’s Medium (DMEM), Penicillin-Streptomycin, Fetal Bovine Serum (FBS), arachidonic acid (AA), LPS derived from *Escherichia coli* 026:B6, indomethacin, acetylsalicylic acid (aspirin or ASA), ibuprofen, bezafibrate, and benzenesulfonamide were from Sigma Aldrich, Milan, Italy. AA520 was synthesized as previously reported (Ammazzalorso et al., 2016), starting from bezafibrate and benzenesulfonamide as starting materials. The Lux 3 μm Amylose-1, 150 mm × 3.0 mm chromatographic column was from Phenomenex, Torrance, United States and the ACQUITY UPLC^®^ BEH C18 1.7 µm chromatographic column was from Waters SpA, Milan, Italy. The Bradford protein assay, β-Mercaptoethanol, the PVDF membrane, and the non-fat milk for immunoblot were from Bio-Rad, Milan, Italy. The anti-GAPDH monoclonal antibody (#sc-47724) and the Ficoll-Paque PLUS density gradient media were from Santa Cruz Biotechnology (Dallas, United States). Colon cancer cell line HCA7 colony 29 (HCA7) was from the European Collection of Cell Cultures (ECC, Salisbury, United Kingdom).

### 2.2 Subjects

Peripheral venous blood samples were drawn from healthy volunteers (n = 10, 7 females, 23–50 years) when they had not taken any non-steroidal anti-inflammatory drug (NSAID) during the 2 weeks preceding the study. This study was carried out following the recommendations of the Declaration of Helsinki after approval by the local Ethics Committee of “G. d’ Annunzio” University of Chieti-Pescara (#254), and informed consent was obtained from each subject.

### 2.3 Effect of AA520 on whole blood COX-1 and COX-2 activities *in vitro*

The compound AA520 was dissolved in DMSO; then 2-µL aliquots of the vehicle or the different solutions of AA520 were added directly into glass test tubes to give the final concentrations of 0.01–300 µM. Duplicate 1-mL aliquots of whole blood drawn from the healthy volunteers were immediately transferred into glass tubes and allowed to clot at 37°C for 1 h. After incubation, serum was immediately separated by centrifugation (1,560 g, 10 min at 4°C) and stored at −80°C until assayed for TXB_2_, which reflects platelet COX-1 activity (Patrono et al., 1980) by using a validated immunoassay (Patrignani et al., 2014) (Cayman Chemical, item#501020). At the same time, 2-µL of the vehicle or the different solutions of AA520 were added to duplicate aliquots of heparinized whole blood samples to give the final concentrations of 0.001–300 µM in the presence of LPS (10 μg/mL) for 24 h as previously described (Patrignani et al., 1994). The contribution of platelet COX-1 was suppressed by adding aspirin *in vitro* at a concentration of 50 μM, solubilized in methanol, and then evaporated through the speed-vac before adding LPS and test-compound. Plasma was separated by centrifugation and kept at −80°C until assayed for PGE_2_ levels by using a specific radioimmunoassay (RIA) (Patrignani et al., 1994). Some experiments were performed to test the effect of bezafibrate and benzenesulfonamide (the starting compounds of the synthesis of AA520) on LPS-stimulated whole blood at the final concentrations of 10–300 μM.

### 2.4 Effects of AA520 on eicosanoid biosynthesis in LPS stimulated whole blood

In LPS-stimulated whole blood, 12R-HETE, 12S-HETE, 15R-HETE, 15S-HETE, 5R-HETE, 5S-HETE, 8R-HETE, 8S-HETE, LTB_4_ and 15R-LXA_4_, were assessed by a modified LC-MS/MS method (Mazaleuskaya et al., 2018). Briefly, samples were extracted by using a liquid-liquid extraction (Maskrey et al., 2007; Tacconelli et al., 2020a): to 0.3 mL of the sample, phosphate buffer (PBS) was added to give 1 mL; then 2.5 mL of a mixture of acetic acid/isopropanol/hexane (2:20:30, v/v/v) and internal standards (d_8_-12S-HETE, d_8_-15S-HETE, d_8_-5SHETE, d_4_-TXB_2_ at the final concentration of 5 ng/mL) were added. The extraction was performed by adding 5 mL of *n*-hexane. Then, the samples were centrifuged at 1,500 *g* at 4°C for 5 min. The dried hexane phases were stored at −80°C until LC-MS/MS analysis. Before analysis, dried lipids were resuspended in 0.2 mL of methanol and analyzed by LC-MS/MS as previously described (Tacconelli et al., 2020a). The LC-MS/MS system consisted of ACQUITY UPLC I-Class/Xevo TQS micro IVD System (Waters) equipped with a Z-Spray ESI source under negative ionization conditions. Deuterated and non-deuterated standards (from Cayman Chemical) were analyzed in MS/MS mode to examine the collision-induced fragmentation spectrum to select specific fragments monitored for each eicosanoid (Hofling et al., 2022). Separation of 12R-HETE, 12S-HETE, 15R-HETE, 15S-HETE, 5R-HETE, 5S-HETE, 8R-HETE, 8S-HETE, 15R-LXA_4_, LTB_4_, PGE_2_ and TXB_2_ was performed using a chiral chromatographic column (Lux 3 μm Amylose-1, 150 mm × 3.0 mm; Phenomenex, Torrance, CA, United States) eluting a 20-min gradient of 50%–100% solvent B (60% methanol, 40% ACN, 0.1% glacial acetic acid) and solvent A (75% water, 25% ACN, 0.1% glacial acetic acid): 50% solvent B for 5 min; 50%–60% solvent B for 4 min; 60%–80% solvent B for 2 min; 80%–90% solvent B for 2 min; 90%–100% solvent B for 1 min, 100% solvent B for 2 min and 50% of solvent B from 17 to 20 min with a flow rate of 0.2 mL/min). The linear standard curves were obtained by adding constant amounts of internal standards to eight different concentrations of each analyte (0.01–500 ng/mL), then the calibration curves were constructed by linear regression of the ratio of the peak areas of the analytes to the areas of the corresponding internal standards. For 8R- and 8S-HETE and 15R-LXA_4_, we used d_8_-12S-HETE as their internal standard (Tacconelli et al., 2020a). The eicosanoid concentrations were calculated by interpolation from the calculated regression lines. The eicosanoid peak areas were extracted and analyzed by using MassLynx software (Waters, United Kingdom). The data were normalized to sample volume and expressed as ng/mL. The detection limit of quantification of each eicosanoid was 10 pg/mL.

### 2.5 Effect of AA520 on COX-1 and COX-2 expression in LPS-stimulated isolated monocytes

Human monocytes were freshly isolated from concentrated buffy coats (obtained from the blood bank of Hospital Renzetti, Lanciano, Chieti, Italy) that were treated *in vitro* with aspirin (50 µM) for 20 min to inhibit the activity of COX-1. As previously described, monocytes were separated from the Ficoll-Paque density gradient media (Patrignani et al., 1994). To characterize the purity of isolated cells, monocytes were incubated with anti-CD14 (1:10) and assessed by FacsVerse cytometer (BD) (Marimuthu et al., 2018). We used four different buffy coats. Assuming an SD of 8 for the % OD values of the COX-1/GAPDH and COX-2/GAPDH immunoreactive bands in LPS-stimulated monocytes, a sample size of 4 would be required to achieve a power of 80% and a significance level of 5% (two-sided) for detecting a difference in means between the LPS-vehicle and AA520 of 20 or more. Cell suspensions routinely contained 90% monocytes (Patrignani et al., 1994). Monocytes (1.5 × 10^6^) grown in RPMI 1640 supplemented with 0.5% FBS, 1% penicillin/streptomycin and 2 mM L-glutamine, were incubated with vehicle (DMSO) or increasing concentrations of AA520 (0.1–10 μM) in the presence of LPS (10 μg/mL) for 24 h. After 24h incubation, monocytes were centrifuged (700 g, 5 min at 4°C); pellets were stored at −80°C until assayed for COX-1 and COX-2 expression by Western blot (Patrignani et al., 2017; Patrignani et al., 1994).

### 2.6 Western blot

COX-1 and COX-2 expression was assessed in monocytes and PPARα in HCA7 cells by a Western blot technique (Patrignani et al., 2017; Patrignani et al., 1994). Briefly, aliquots of cell lysates were loaded onto 9% Sodium Dodecyl Sulphate-PolyAcrylamide Gel Electrophoresis (SDS-PAGE), transferred to PVDF membrane, and blocked with a solution of 5% blotting grade blocker in tris-buffered saline-0.1% Tween-20 (TBS-Tween-20). The membrane was incubated overnight with COX-2 (mouse) monoclonal antibody (#160112, Cayman Chemical), COX-1 ovine polyclonal antibody (#160108, Cayman Chemical), PPARα rabbit polyclonal antibody (#227074, Abcam) and GAPDH monoclonal antibody (#sc-47724, Santa Cruz Biotechnology) used as the loading control. Membranes were developed using ECL Western blotting Detection Reagents. Results were obtained using a digital imaging system Alliance 4.7 (UVITEC, Cambridge, United Kingdom) (Patrignani et al., 2017).

### 2.7 Assessment of the inhibition of COX-2 and its reversibility by AA520 in HCA-7 cells

We studied the mechanism of inhibition of COX-2 by AA520 in comparison to aspirin, indomethacin, and ibuprofen in colon cancer cell line HCA-7 colony 29 (HCA-7), selectively expressing only COX-2. The HCA-7 cell line was from the European Collection of Cell Cultures (ECC, Salisbury, United Kingdom). The HCA-7 cells, used at passage levels 11–18, were cultured in DMEM supplemented with 10% FBS, 1% penicillin/streptomycin, and 1% L-glutamine.

Before each experiment, cells were plated at the concentration of 1 × 10^6^ in 5 cm plates (volume 3 mL) containing 2 mL of DMEM supplemented with 0.50% of FBS for 16 h. First, we assessed the inhibition of COX-2 by AA520 by preincubating the cells with vehicle (DMSO) or with different concentrations of the compound (30 min at room temperature); then, AA (0.5 μM) was added for a further 30 min at 37°C, and supernatants were collected and assessed for PGE_2_ levels by RIA.

The kinetics of the interaction of AA520 and other NSAIDs with COX-2 was assessed by performing biochemical studies (Vitale et al., 2013) evaluating the residual inhibition of PGE_2_ biosynthesis by HCA7 cells after extensive washing of the cells versus the values obtained without washing. Briefly, AA520, indomethacin (a time-dependent inhibitor of COX), aspirin (ASA, an irreversible inhibitor of COX), and ibuprofen (a reversible inhibitor of COX) were incubated with the cells at a concentration of 100 μM for 30 min at room temperature. In some experiments, AA (0.5 μM) was added, and the incubation continued for 30 min at 37°C. In other experiments, cells preincubated with the different compounds were washed three times with 3 mL of DMEM (without FBS), resuspended with medium (without FBS), and stimulated with AA, 0.50 μM for 30 min at 37°C. In both experimental conditions (without or with washing passages), PGE_2_ production was determined in the medium by RIA as an index of COX-2 activity (Patrignani et al., 1994). After trypsinization and centrifugation, protein quantification was performed using the Bradford method.

### 2.8 Development of an LC-MS/MS method for the qualitative assessment of AA520, bezafibrate, and benzenesulfonamide

We have developed a LC-MS/MS method in “Multiple Reaction Monitoring (MRM)” mode (LC/MS/MRM) which allowed qualitative analysis of AA520, bezafibrate and benzenesulfonamide by using an ACQUITY UPLC I-Class/Xevo TQS micro IVD System (Waters) equipped with a Z-Spray ESI source under negative ionization conditions. The three compounds were solubilized in methanol at a final concentration of 1,000 ng/mL and infused into the electrospray ionization source (ESI ZSpray), under negative ionization conditions, at a rate of 50 μL/min, to obtain the MS and MS/MS fragmentation spectra.

Chromatographic separation of the three compounds was performed using an ACQUITY UPLC^®^ BEH C18 1.7 µm chromatography column (Waters) with the following mobile phases: A) water (0.1% FA); B) ACN (0.1% FA). The mobile phases eluted with a flow rate of 0.3 mL/min according to the following gradient: 0–1 min: 100%A; 1–7 min: 10%A; 7–9 min:100%A. The volume injected was 5 µL.

### 2.9 Qualitative evaluation of AA520, bezafibrate, and benzenesulfonamide by LC-MS/MS in whole blood incubated for 24 h at 37°C with AA520

Aliquots (1 mL) of heparinized whole blood were incubated with AA520 at 37°C for 24 h. At the end of the incubation, the plasma was separated by centrifugation (10 min at 1560 g at 4°C). Then aliquots of 200 µL of plasma were extracted with 1 mL of acetonitrile (Saraner et al., 2019); after vortexing for 30 s, the samples were centrifuged at 1800 *g* for 10 min. Finally, 5 µL of the supernatant was injected into the LC-MS/MS system to determine the presence of AA520 or potential metabolites.

### 2.10 MTT assay

HCA7 cells were seeded at 4 × 10^3^ cells/well in DMEM supplemented with 0.5% FBS and 1% penicillin/streptomycin at 37°C. Then, cells were treated with AA520 (1–10 μM), rofecoxib (10 μM), GW6471 (a PPARα antagonist, 10 and 25 μM), or vehicle (DMSO), and the viability was assessed up to 72 h of exposure by using the [(3-(4,5-dimethylthiazol-2-yl)-2,5-diphenyltetrazolium bromide] (MTT) assay according to the manufacturer’s instructions (CyQUANT™ MTT Cell Viability Assay, Invitrogen).

### 2.11 Molecular modeling

#### 2.11.1 Protein and ligand preparation

The 2.4 Å resolution X-ray structure of murine COX-2 in complex with celecoxib (PDB 3LN1) (Wang et al., 2010) was downloaded from the Protein Data Bank. The structure of murine COX-2 is highly similar to the human enzyme, with 87% identity and strict sequence conservation in the active site (Kurumbail et al., 1996). The Protein Preparation Wizard in Maestro (Protein Preparation Wizard; Epik, Schrödinger, LLC, New York, NY, 2021; Impact, Schrödinger, LLC, New York, NY, 2021; Prime, Schrödinger, LLC, New York, NY, 2021) was used to prepare the selected structure for docking studies: all the crystallographic water molecules and other chemical components were removed; the right bond orders, charges, and atom types were assigned; and the hydrogen atoms were added. The H-bond network was optimized by exhaustive sampling of rotamers, tautomers, and protonation states of titratable amino acids at neutral pH. Finally, a restrained minimization was performed on the protein structures using the Impref module, by imposing a 0.3 Å RMSD limit from the initial coordinates as constraint. The *in-house* small library of compounds, including AA520 (Ammazzalorso et al., 2016), was prepared for *in silico* studies with LigPrep (LigPrep, Schrödinger, LLC, New York, NY, 2021) in order to generate suitable 3D conformations and tautomerization states at pH 7.

#### 2.11.2 Docking calculations

The virtual screening of the *in-house* library of compounds was accomplished by using Glide (Glide, Schrödinger, LLC, New York, NY, 2021) (Friesner et al., 2004; Halgren et al., 2004) in SP mode.

Docking of AA520 was carried out with the Glide Induced Fit Docking (IFD) protocol (Glide, Schrödinger, LLC, New York, NY, 2021; Prime, Schrödinger, LLC, New York, NY, 2021) (Farid et al., 2006; Sherman et al., 2006a; Sherman et al., 2006b). For both virtual screening and IFD, the docking grid was generated by considering an inner box of 10 Å × 10 Å × 10 Å and an outer box of 30 Å × 30 Å × 30 Å surrounding the bound celecoxib. In the case of IFD, an extended sampling protocol was adopted, which returns up to 80 poses: in the first stage, docking is conducted using a softened potential and removal of side chains, on the basis of solvent-accessible surface areas and B-factors. The results of this procedure are clustered to obtain representative poses. Then, for residues within 5 Å of any ligand pose, a Prime side-chain prediction is carried out, followed by minimization of both residues and ligand. Finally, the ligand is re-docked, using default Glide SP settings, into the induced-fit receptor structure, and each output pose is scored. Both the Glide Emodel and GlideScore lowest-energy values were considered for final pose selection. Before proceeding with the docking simulations of the compound under study, we investigated pose generation quality by re-docking the co-crystalized celecoxib (PDB 3LN1). The above-described IFD protocol well reproduced the experimental geometries, with RMSD value of 0.48 Å.

#### 2.11.3 Molecular dynamics simulations

The protein-ligand complex obtained by the above-described IFD approach was selected for molecular dynamics simulations, carried out by means of Desmond (Bowers et al., 2006). Briefly, the system was solvated in a 10 Å layer orthorhombic box using TIP3P water model, and then neutralized by adding counterions. A salt concentration of 0.15 M of NaCl was also included in the simulation box to reproduce the physiological conditions. OPLS_2005 (Jorgensen et al., 1996) was used as force field. The system was relaxed before the simulation by using the protocol implemented in Desmond; then, the simulation was run for 100 ns under a NTP ensemble using the Nose-Hoover thermostat to maintain a constant temperature of 300 K and Martyna-Tobias-Klein barostat to maintain the pressure at 1 atm. The trajectories were saved at 100 ps intervals for analysis. The obtained trajectory was clustered according to the RMSD matrix of a specified set of atoms (backbone) by employing “Desmond Trajectory Clustering”, which uses an affinity propagation clustering method (Frey and Dueck, 2007). A trajectory frame extraction interval of 10 and a maximum output number of clusters to 10 were set. A total of 15 clusters were obtained (Supplementary Table S1), of which the representative structure from the most populated cluster was selected for subsequent analysis. The “Simulation Interactions Diagram” tool was then used for post-MD analysis. The stability of MD simulations was monitored by observing the root mean square deviation (RMSD) of the ligand and protein atom over simulation time.

The representative structure obtained from the clustering procedure was then used to run the calculation of Prime MM-GBSA (Prime, Schrödinger, LLC, New York, NY, 2021). This method can be used to approximate the free energy of binding between a protein and a ligand. The calculations employed predefined dielectric constants, the OPLS_2005 force field, and the VSGB solvation model (Li et al., 2011). A more negative value indicates stronger binding. The obtained values of ΔG_bind_ were compared with those calculated using as reference the COX-2/celecoxib structure obtained from the protein preparation procedure (see above).

All the figures were rendered with PyMOL (The PyMOL Molecular Graphics System, Version 2.0 Schrödinger, LLC).

### 2.12 Statistical analysis

The data have been reported as mean ± standard error of the mean (SEM) or standard deviation (SD) as specified. The statistical analysis was performed using GraphPad Prism software (version 10.00 for Mac; GraphPad, San Diego, CA). The values of P < 0.05 were considered statistically significant. The specific statistical text used in each experiment is reported in the Figure legends. In the experiments assessing the % inhibition of PGE_2_ in LPS-stimulated whole blood by AA520, the concentration of PGE_2_ produced in LPS-stimulated whole blood was subtracted from that produced without LPS (baseline) The concentration-response curves were obtained using GraphPad Prism software (version 10.00 for Mac). GraphPad Prism software obtained the IC_50_ and 95% confidence interval (CI) values of the sigmoidal concentration-response data.

## 3 Results

### 3.1 Identification of COX-2 inhibitors by virtual screening

We virtually screened the in-house small library of compounds described previously (Ammazzalorso et al., 2016) to find novel small molecules targeting PPARα and COX-2. Virtual screening calculations were performed using Glide on COX-2 3D structure in complex with celecoxib (PDB 3LN1). Virtual screening results were sorted based on the docking scores and visual inspection (a more detailed description of the COX-2 binding site is reported in section 3.7). Compounds **1b**, **1e,** and **2b** could not be proficiently docked within the COX-2 active site and were discarded from our analysis. Then, we focused on the top-scoring compounds endowed with potent PPARα antagonistic activity, namely **1d**, **2b**, **6** (hereafter AA520), and **4** (Supplementary Table S2). Finally, we decided to prioritize compound AA520 because of its exquisite selectivity on PPARα concerning PPARγ (Ammazzalorso et al., 2016). In fact, the clinical candidate TPST-1120 possesses high specificity (>250-fold) for PPARα over the other isoforms (Stock et al., 2017).

### 3.2 Effect of AA520 on eicosanoid generation in human whole blood

In human whole blood allowed to clot at 37°C for 1 h, TXB_2_ is generated in serum, and it is mainly derived from platelets in response to endogenously formed thrombin (Patrono et al., 1980). It represents an index of the maximal capacity of platelet COX-1 to generate this prostanoid. Serum TXB_2_ at baseline averaged 421 ± 205 ng/mL (n = 10; mean ± SD). In heparinized human whole blood samples, incubated with LPS (10 μg/mL) at 37°C for 24 h, PGE_2_ was generated and averaged 19.6 ± 9.8 ng/mL (n = 10; mean ± SD). Under these experimental conditions, it was previously reported that LPS induces COX-2 expression in leukocytes in a time-dependent fashion, and PGE_2_ paralleled the COX-2 expression (Patrignani et al., 1994). Aspirin (50 μM) was added at the beginning of the incubation to prevent the contribution of platelets to the generation of PGE_2_. Aspirin causes irreversible inhibition of platelet COX-1 that persists throughout the 24 h of incubation due to the limited capacity of the anucleated platelet to *de novo* protein synthesis (Evangelista et al., 2006). Aspirin is unstable in plasma and is metabolized to salicylic acid (a weak COX inhibitor) before the induction of COX-2 in leukocytes in response to LPS (Cipollone et al., 1997). Thus, under these experimental conditions, aspirin does not interfere with the activity of COX-2. In unstimulated heparinized whole blood, the PGE_2_ levels were 0.34 ± 0.22 ng/mL (n = 10). The assessment of serum TXB_2_ and LPS-induced PGE_2_ in whole blood is considered the gold standard assay to assess the selectivity of NSAIDs towards COX-2. As shown in Figures 1B, C, AA520 inhibited LPS-induced PGE_2_ in a concentration-dependent fashion with an IC_50_ of 0.10 μM (95% CI: 0.05–0.23). The compound only marginally inhibited platelet COX-1 activity at the maximum concentration of 300 μM. The COX-1/COX-2 IC_50_ ratio was >697.

### 3.3 Targeted lipidomics of LPS-stimulated human whole blood

**FIGURE 2:**
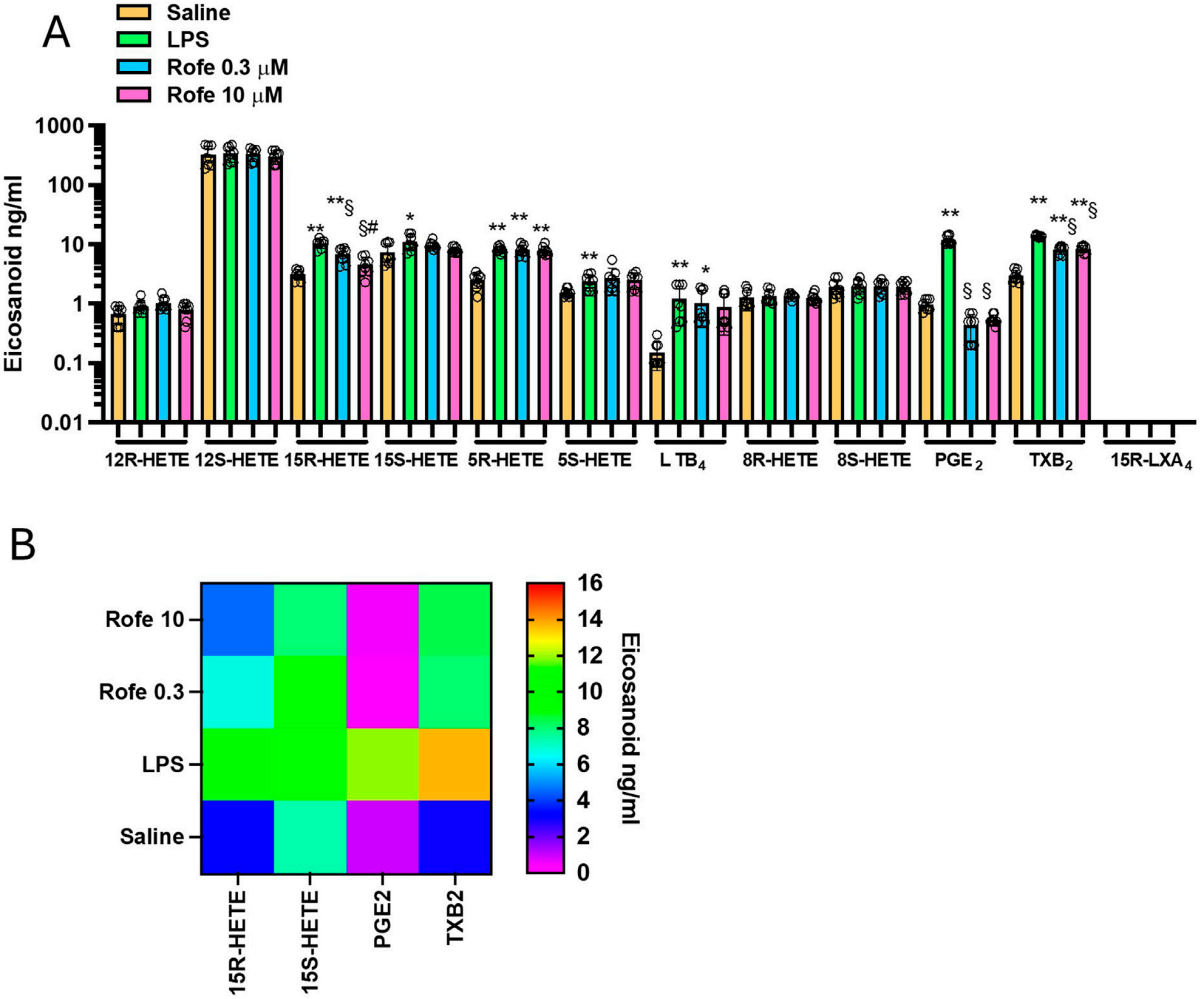
Effects of rofecoxib on eicosanoid biosynthesis in LPS-stimulated-whole blood by targeted lipidomics. **(A)** Aliquots (1 mL) of heparinized whole blood drawn from healthy volunteers are incubated with rofecoxib (0.3 and 10 μM) or DMSO (vehicle of rofecoxib) in the presence of NaCl (0.9% w/v, named saline) or LPS (10 μg/mL, dissolved in NaCl 0.9% w/v) for 24 h; after centrifugation, eicosanoid levels (12R-HETE, 12S-HETE, 15R-HETE, 15S-HETE, 5R-HETE, 5S-HETE, 8R-HETE, 8S-HETE, LTB_4,_ PGE_2_, TXB_2_ and 15R-LXA_4_) were analyzed by using LC-MS/MS. Results are depicted as ng/mL of each eicosanoid [mean ± SEM, n = 8 (5 females and 3 males), and individual values were also reported]. For each eicosanoid, we used one-way ANOVA and Dunnett *post hoc* test (to compare the means of different treatments versus saline or LPS), *P < 0.05, **P < 0.001, versus saline, §P < 0.01 versus LPS; or one-way ANOVA and Tukey *post hoc* test (to compare the mean of each column with the mean of every other column), #P < 0.05, versus 0.3 μM (15R-HETE); **(B)** Heat map of 15R-HETE, 15S-HETE, PGE_2,_ and TXB_2_ (mean values) in saline, LPS and Rofecoxib (0.3 and 10 μM) conditions.

To verify the impact of AA520 on different enzymatic and nonenzymatic pathways of AA metabolism, we modified a previously published LC-MS/MS method (Mazaleuskaya et al., 2018). To assess 5-lipooxygenase (LOX) activity, we measured 5S-HETE and LTB_4_; for the 12S-LOX activity, we assessed 12S-HETE; for 15-LOX-1 activity, we evaluated 15S-HETE and 12S-HETE; for COX-1 and COX-2 activity we measured PGE_2_, TXB_2_, 15R-HETE and 15S-HETE (these HETEs are minor products of COX activity) (Powell and Rokach, 2015; Mazaleuskaya et al., 2016; 2018; Contursi et al., 2022). We also measured 5R-HETE, 8S-HETE, 8R-HETE, and 12R-HETE as markers of nonenzymatic oxidation of AA. Finally, we assessed 15R-LXA_4_ (also named aspirin-triggered LXA_4_), a product of 15R-HETE and 5-LOX (Serhan, 2002). As shown in Figure 2A and Supplementary Table S3, LPS significantly increased PGE_2_, TXB_2_, 15R-HETE, 15S-HETE, 5S-HETE, LTB_4_, and 5R-HETE vs. unstimulated human whole blood. Noteworthy, 15R-LXA_4_ was undetectable (i.e., <10 pg/mL) in unstimulated and LPS-stimulated whole blood.

### 3.4 Comparison of the effects of rofecoxib and AA520 on targeted lipidomics of LPS-stimulated human whole blood

As shown in Figures 2A, B, the selective COX-2 inhibitor rofecoxib (Patrono et al., 2001; Tacconelli et al., 2002) significantly reduced PGE_2_, TXB_2_, and 15R-HETE, while the other eicosanoids were not significantly affected. However, the extent of reduction of TXB_2_ and 15R-HETE was lower than PGE_2_. At 10 μM of rofecoxib, TXB_2_, 15R-HETE, and PGE_2_ reduction were 39, 56, and 95%, respectively. The lower inhibition of TXB_2_ vs. PGE_2_ is because TXB_2_ can also be generated from leukocyte COX-1. The contribution of platelet COX-1 is excluded since aspirin was added at the beginning of the incubation. 15R-HETE is produced due to AA’s different conformational interaction in the COX active site (Thuresson et al., 2001; 2002; Powell and Rokach, 2015), and rofecoxib may be less effective in competing with AA in this conformation.

**FIGURE 3:**
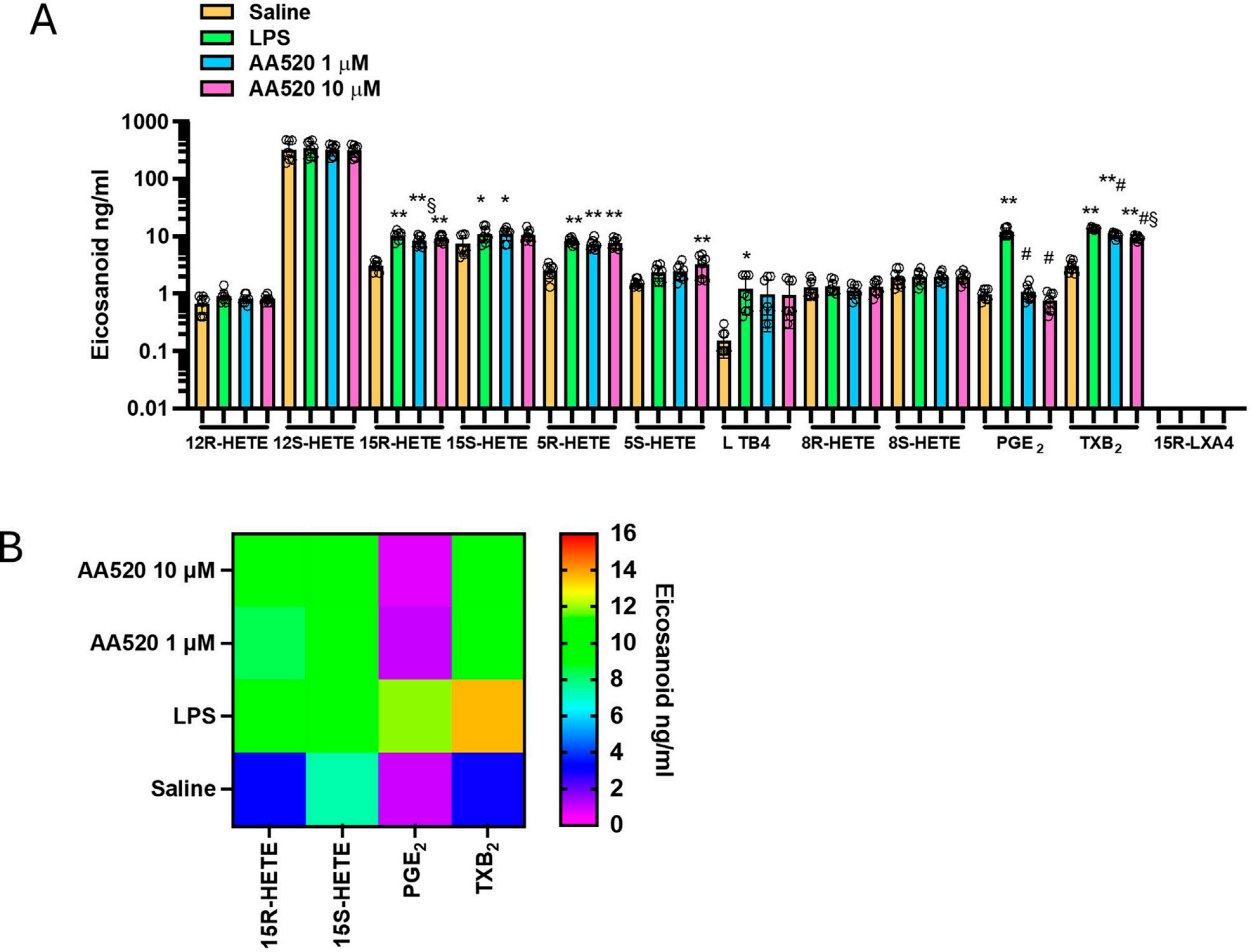
Effects of AA520 on eicosanoid biosynthesis in LPS-stimulated whole blood by targeted lipidomics. **(A)** Aliquots (1 mL) of heparinized whole blood drawn from healthy volunteers are incubated with AA520 (1 and 10 μM) or DMSO (vehicle of AA520) in the presence of NaCl (0.9% w/v, named saline) or LPS (10 μg/mL, dissolved in NaCl 0.9% w/v) for 24 h; after centrifugation, eicosanoid levels (12R-HETE, 12S-HETE, 15R-HETE, 15S-HETE, 5R-HETE, 5S-HETE, 8R-HETE, 8S-HETE, LTB_4_ PGE_2_, TXB_2_ and 15R-LXA_4_) were analyzed by using LC-MS/MS. Results are depicted as ng/mL of each eicosanoid (mean ± SEM, n = 8, five females and 3 males). For each eicosanoid, we used one-way ANOVA and Dunnett *post hoc* test (to compare the means of different treatments versus saline or LPS), *P < 0.05, **P < 0.01, versus saline, #P < 0.001 versus LPS; or one-way ANOVA and Tukey *post hoc* test (to compare the mean of each column with the mean of every other column), §P< 0.05 versus 10μM; **(B)** Heat map of 15R-HETE, 15S-HETE, PGE_2,_ and TXB_2_ (mean values) in saline, LPS and AA520 (1 and 10 μM) conditions.

Next, we tested AA520 on the generation of eicosanoids in LPS-stimulated whole blood (Figures 3A, B). Similarly to rofecoxib, the compound significantly reduced PGE_2_, TXB_2,_ and 15R-HETE, while the other eicosanoids were unaffected. The extent of reduction of TXB_2_ and 15R-HETE was lower than PGE_2_. At 1 μM of AA520, TXB_2_, 15R-HETE, and PGE_2_ reduction were 23, 19, and 91%, respectively.

**FIGURE 4:**
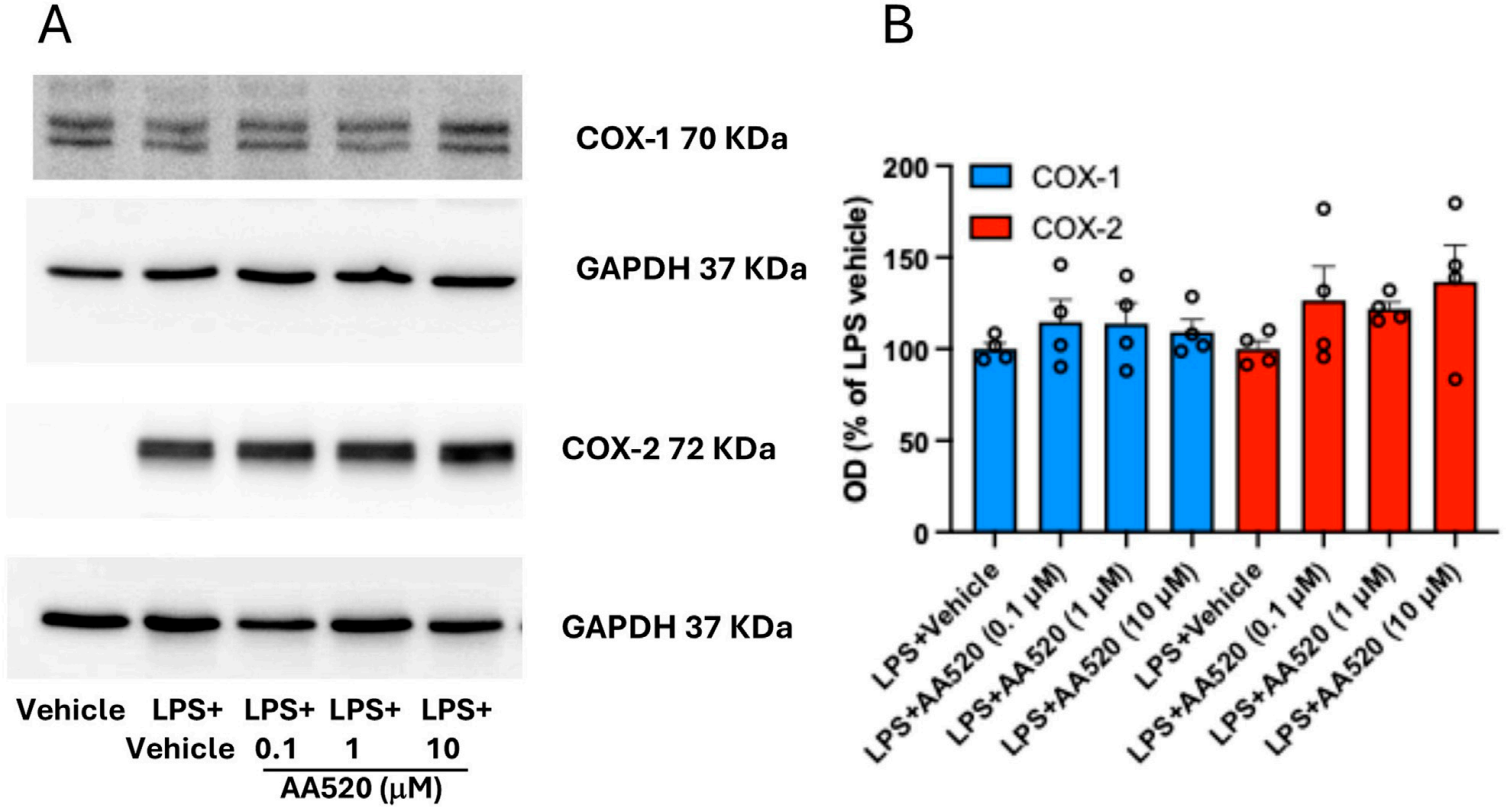
Effect of AA520 on COX-1 and COX-2 expression in LPS-stimulated-isolated monocytes. Human monocytes were freshly isolated from concentrated buffy coats. Monocytes (1.5 × 10^6^) grown in RPMI 1640 supplemented with 0.5% FBS, 1% penicillin/streptomycin and 2 mM L-glutamine, were incubated with vehicle (DMSO + NaCl 0.9% w/v) without or with LPS (final 10 μg/mL; vehicle LPS); monocytes were also incubated with LPS in the presence of increasing concentrations of AA520 (0.1–10 μM, dissolved in DMSO) at 37°C for 24 h. After 24 h of incubation, monocytes were centrifuged, and pellets were assayed for COX-1 and COX-2 expression by Western blot **(A)**. The optical density (OD) ratio values of COX-1 and COX-2 immunoreactive bands versus GAPDH bands detected in monocytes treated with LPS vehicle were reported as % of the mean; the effect of increasing concentrations of AA520 (0.1–10 μM) on monocyte COX-1/GAPDH or COX-2/GAPDH were reported as % of LPS vehicle value of each experiment **(B)**. The data were analyzed using one-way ANOVA and Dunnett *post hoc* test (to compare the means of different treatments versus LPS vehicle). The OD values of COX-1/GAPDH or COX-2/GAPDH detected in LPS vehicle were reported as % of the mean + SEM, n = 4.

In LPS-isolated human monocytes, AA520 did not significantly affect the protein expression of COX-1 and COX-2 (Figures 4A, B).

Altogether, these findings show that AA520 is a highly selective inhibitor of COX-2 activity.

### 3.5 Stability of AA520 in LPS-stimulated whole blood and effects of bezafibrate and benzenesulfonamide on PGE_2_ generation

**FIGURE 5:**
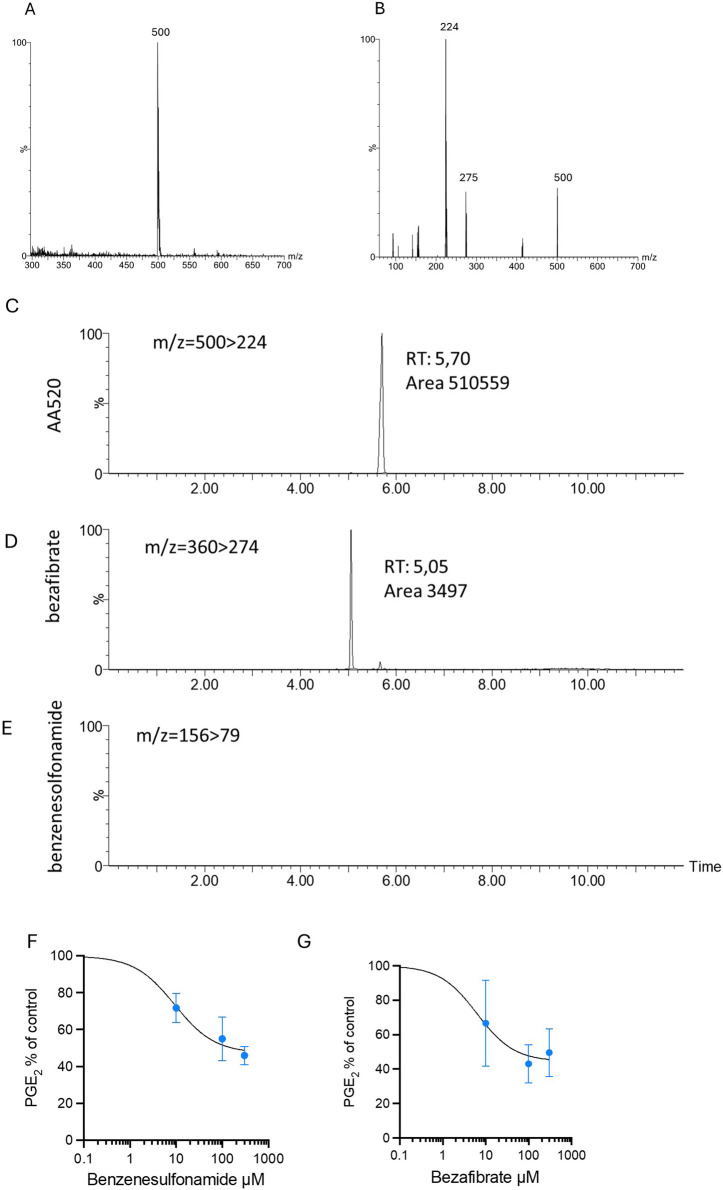
Stability of AA520 in LPS-stimulated whole blood and effects of bezafibrate and benzensulfonamide on PGE_2_ generation. **(A, B)** Development of a method for the qualitative analysis of AA520: MS **(A)** and MS/MS fragmentation **(B)** spectra of AA520 by triple quadrupole mass spectrometry (MS). AA520 is solubilized in methanol at a final concentration of 1,000 ng/mL and infused into the electrospray ionization source (ESI Z-Spray) under negative ionization conditions at a rate of 50 μL/min. In **(B)**, it is shown the fragmentation spectrum of AA520, obtained with a collision energy of 15 eV; **(C–E)** qualitative evaluation of AA520, bezafibrate, and benzenesulfonamide by LC-MS/MS in whole blood samples incubated for 24 h at 37°C with AA520 (100 μM); a one-mL aliquot of whole blood was incubated with AA520 (100 μM) for 24h at 37°C and after centrifugation and extraction, the sample was injected into the LC-MS/MS system to determine the presence of AA520 and its potential metabolites bezafibrate and benzenesulfonamide; the chromatographic profile of their main fragments m/z 500 > 224 for AA520, m/z360 > 274 for bezafibrate and m/z 156 > 79 for benzenesulfonamide are shown. **(F, G)** Concentration-response curves of inhibition of LPS-induced-PGE_2_ biosynthesis by benzenesulfonamide and bezafibrate; increasing concentrations of benzenesulfonamide **(F)** and bezafibrate **(G)** (10–300 µM) or vehicle (DMSO + NaCl 0.9% w/v) were incubated with heparinized whole blood samples, withdrawn from healthy volunteers, after suppressing the contribution of platelet COX-1 by adding aspirin (50 µM) *in vitro* solubilized in methanol and then evaporated, in the presence of LPS (10 μg/mL) for 24 h; after centrifugation, PGE_2_ levels were analyzed as an index of LPS-induced-COX-2 activity, by specific immunoassay; results are depicted as percent of control (LPS vehicle) (mean ± SEM, n = 3, 2 females and 1 male).

Since AA520 was synthesized from the starting products bezafibrate and benzenesulfonamide, we studied the purity of the compound, and a chromatographic LC-MS/MS method was applied to AA520 and its starting compounds bezafibrate and benzenesulfonamide. Figure 5, panels A and B, show the MS and the fragmentation spectra of AA520, respectively. The MS spectrum of AA520 did not display bezafibrate and benzenesulfonamide ions (m/z 360 and m/z 156, respectively, Figure 5A), supporting its purity. From the fragmentation spectra of AA520 (Figure 5B), we have chosen the most abundant fragment to follow for the qualitative analysis of AA520, i.e., m/z 500 > 224 (Figure 5B). For bezafibrate and benzenesulfonamide, the most abundant fragments were m/z360 > 274 and m/z 156 > 79, respectively (not shown).

We assessed the possible metabolization of AA520 (100 μM) to bezafibrate and benzenesulfonamide in heparinized human whole blood incubated for 24 h at 37°C. At the end of the incubation, plasma samples were analyzed for AA520, bezafibrate, and benzenesulfonamide by LC-MS/MS (Figures 5C–E). We detected only tiny amounts of bezafibrate (0.68% of AA520), while benzenesulfonamide was undetectable (<0.1 μM).

We assessed whether benzenesulfonamide and bezafibrate affected COX-2 activity in LPS-stimulated whole blood. As shown in Figure 5, panels F and G, benzenesulfonamide, and bezafibrate reduced PGE_2_ generation incompletely (approximately 50%), even at the high concentration of 300 μM.

These data suggest that AA520 is stable in blood up to 24 h and that the possible formation of approximately 1% of bezafibrate did not contribute to COX-2 inhibition by AA520.

### 3.6 Assessment of the mechanism of inhibition of COX-2 by AA520 in the human colon cancer cell line HCA7

**FIGURE 6:**
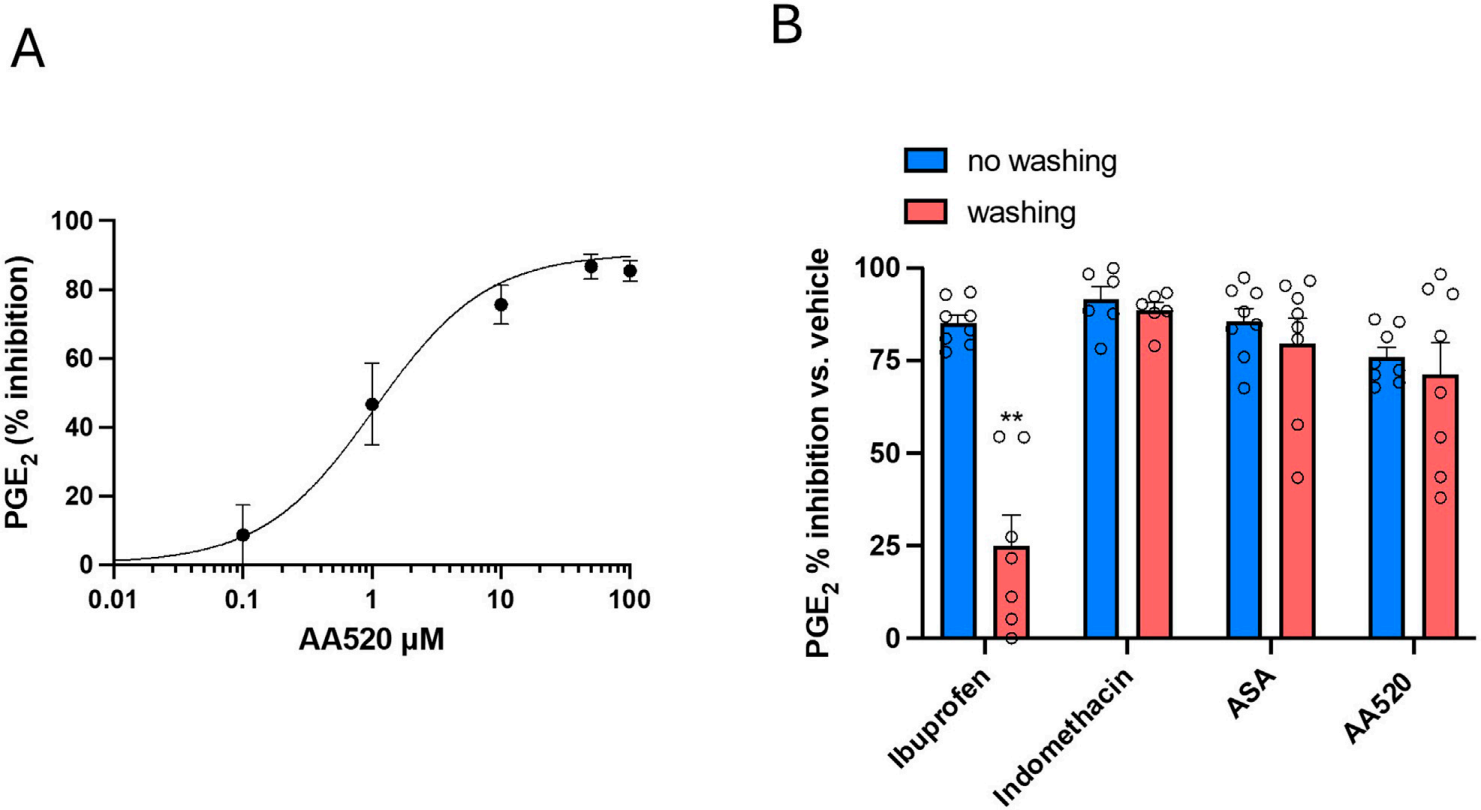
Assessment of the inhibition of COX-2 and its reversibility by AA520 in HCA-7 Cells. **(A)** Concentration-dependent inhibition of PGE_2_ biosynthesis by AA520 in HCA-7 colony 29 cell line (HCA-7 cells). HCA-7 cells were incubated with DMSO or increasing concentrations of AA520 (0.01–100 μM) for 30min; then, cells were incubated with AA (0.5 μM) for a further 30 min at 37°C, and the levels of PGE_2_ were assessed in the conditioned medium by a validated immunoassay. Data are reported as mean ± SEM, n = 4, and represented as % inhibition of PGE_2_ generated without the compounds (vehicle). **(B)** Kinetics of the interaction of AA520 and other NSAIDs on COX-2 of HCA-7 cells; the inhibition of COX-2-dependent PGE_2_ biosynthesis was assessed by preincubating HCA-7 cells with AA520 (10 μM), indomethacin (a time-dependent inhibitor of COX, 100 μM), aspirin (acetylsalicylic acid, ASA, 100 μM) (an irreversible inhibitor of COX, 100 μM), or ibuprofen (a reversible inhibitor of COX, 100 μM) for 30 min; then, AA (0.5 μM) was added, and the incubation continued for 30 min at 37°C. In other experiments, cells preincubated with the different compounds were washed three times with 3 mL of DMEM (without FBS), resuspended with medium (without FBS), and stimulated with AA, 0.50 μM for 30 min at 37°C. In both experimental conditions (without or with washing passages), PGE_2_ production was determined in the medium by a validated immunoassay as an index of COX-2 activity. Data are shown as % inhibition (versus vehicle), mean ± SEM, n = 6–8. The data were analyzed using a two-way ANOVA and Šídák’s multiple comparisons test; **P< 0.01 versus no washing condition.

As previously reported (Hofling et al., 2022; Tacconelli et al., 2020b), HCA7 cells express COX-2 but not COX-1. We studied the concentration-dependent inhibition of COX-2-dependent PGE_2_ biosynthesis by AA520 in HCA7 cells stimulated with 0.5 μM of AA. As shown in Figure 6A, AA520 inhibited in a concentration-dependent fashion COX-2-dependent PGE_2_ with an IC_50_ of 1.05 (95% CI: 0.58–1.97) μM.

We determined the kinetics of the interaction of AA520 on HCA7 cell COX-2. This involved assessing whether the interaction is rapidly reversible, time-dependent reversible, or irreversible. To achieve this, we compared the extent of PGE_2_ biosynthesis inhibition in cells exposed to the compound for 30 min and subsequently washed versus those not washed. Similar experiments were performed with ASA, an irreversible inhibitor of COX; indomethacin, a time-dependent/slowly reversible inhibitor of COX; and ibuprofen, a time-independent/rapidly reversible inhibitor of COX (Walker et al., 2001; Blobaum and Marnett, 2007; Vitale et al., 2013). As shown in Figure 6B, the inhibition of COX-2 activity by AA520 was not significantly affected by the washing of cells similar to ASA and indomethacin.

In contrast, washing the cells almost completely reversed the inhibition of ibuprofen. These results suggest that AA520 tightly interacts with COX-2, resembling the mechanism of indomethacin, i.e., slowly reversible inhibition. However, these results cannot exclude an irreversible interaction with the enzyme similar to the mechanism of inhibition by ASA. To clarify this issue, we performed docking studies.

### 3.7 Molecular basis of AA520 inhibitory activity on COX-2

To elucidate the molecular basis of the activity of AA520, computational studies were performed using the crystal structure of COX-2 in complex with celecoxib (PDB 3LN1) (Wang et al., 2010).

**FIGURE 7:**
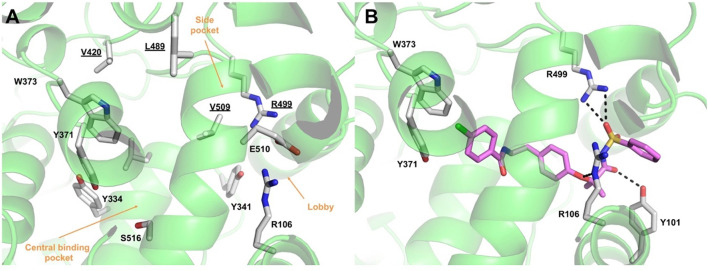
**(A)** Overview of the COX-2 (green ribbons) active site. Amino acids lining the different pockets are shown as white sticks and labeled. Amino acids lining the COX-2 “side pocket” are underlined. **(B)** Binding mode of compound AA520 (violet sticks) into COX-2 (green ribbons, PDB 3LN1), as predicted by IFD calculations. Only amino acids involved in pivotal contacts are displayed (white sticks) and labeled. H-bonds discussed in the text are depicted as dashed black lines.

The COX active site comprises a predominantly hydrophobic channel that penetrates deeply into the catalytic domain. Although amino acid numbering for COX-1 is usually applied to COX-2, herein, we will retain the numbering of the selected COX-2 X-ray structure (PDB 3LN1). It is worth noting that the numbers of the amino acids in COX-2 are lower by 14 than those of the corresponding residues in COX-1. For instance, the catalytic tyrosine residue is 385 in COX-1 and 371 in COX-2. Based on AA binding, it is possible to divide the active site into different pockets. Residues R106, Y341, and E510 define a “constriction site” (Figure 7A), which opens up the so-called “lobby” (Rouzer and Marnett, 2020). These residues frequently interact with fatty acids or other polar functional groups of substrates or inhibitors. The central binding pocket, instead, contains the residues directly involved in catalysis (Y334, L338, Y371, W373, G512, and S516). COX-2 is known to have a larger binding cavity (Ahmadi et al., 2022), with a “side pocket” next to the active site comprising the amino acids V509, V420, L489, and R499 compared with COX-1, in which such amino acids are changed to I523, I434, F503, and H513, respectively. In particular, the smaller valine residue V509 in COX-2 is primarily responsible for the larger size of its active site. The side pocket is a key binding site exploited by many COX-2 selective inhibitors, including coxibs.

The results of the IFD approach showed that AA520 fitted well within the COX-2 active site, stabilized by several interactions (Figure 7B; Supplementary Figure S1): the oxygen atom of the sulfonimide moiety was H-bonded with the side chain of R499, whereas the carbonyl oxygen accepted an H-bond from the side chain of Y101. In addition, R106 engaged a salt bridge with the negatively charged nitrogen atom of the sulfonimide moiety and a further H-bond with the oxygen atom of the phenoxy moiety. The ligand’s tail formed mainly hydrophobic interactions, with the distal *p*-chlorobenzoyl moiety establishing π−π stacking interactions with Y371 and W373. As described above, Y371 is a key catalytic residue that, during enzyme activation, donates an atom of hydrogen to heme (Rouzer and Marnett, 2020). On the other hand, W373 has been reported to possess a role in the correct positioning of AA within the active site by mutagenesis studies, suggesting that both steric bulk and hydrophobicity at this position are important (Thuresson et al., 2001).

**FIGURE 8:**
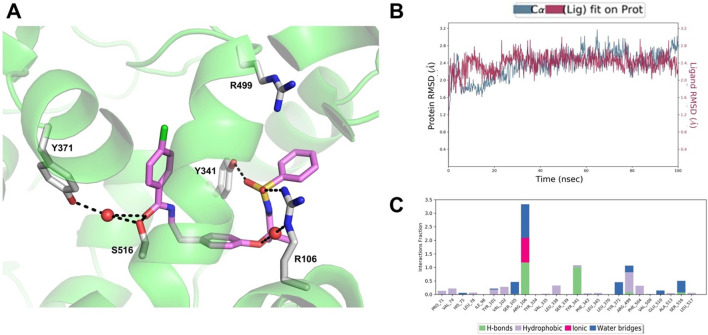
**(A)** Binding mode of AA520 (violet sticks) into COX-2 after 100 ns MD. The representative structure from the most populated cluster is shown. Only amino acids involved in pivotal contacts are displayed (white sticks) and labeled. Waters that engage stable interactions are displayed as red spheres. **(B)** RMSD plot of the protein Ca (blue line) and ligand heavy atoms (red line) with respect to the initial MD frame taken as reference. **(C)** Histogram plot showing the protein interactions with the ligand monitored throughout the simulation.

The results of the MD simulations and clustering carried out on the COX-2/AA520 complex obtained by the IFD approach showed that the compound is well stabilized within the COX-2 binding site, assuming a horseshoe-shaped conformation within the constriction site and the central binding cavity (Figure 8A). The RMSD analysis of both protein and ligand revealed stable trajectories (Figure 8B). AA520 was further stabilized by a water molecule in its interaction with R106; also, a very strong H-bond with Y341 emerged during the simulation. The benzenesulfonamide head group slightly rearranged to form a cation-π interaction with R499, whereas the ligand’s tail group also engaged water-mediated H-bonds with Y371 and S516 through the carbonyl group.

In addition, in order to roughly estimate the binding affinity of AA520 with COX-2, we employed the Prime MMGBSA approach, which provides a useful method to approximate the free energy of binding between a protein and a ligand (Li et al., 2011). The above-described representative structure was used to run the calculation of Prime MM-GBSA; for AA520 we obtained a ΔG_bind_ = −97.20 kcal/mol, suggesting a very favorable binding affinity, considering that for celecoxib we obtained a ΔG_bind_ = −100.53 kcal/mol.

The binding mode of AA520 allowed us to shed some light on the exquisite COX-2 selectivity shown in inhibition assays. A primary determinant for selectivity seems to reside in the interactions formed by the benzenesulfonamide group with R499, which is replaced by histidine in COX-1. This latter would not be able to extend sufficiently to interact with this crucial ligand’s moiety. Worthy of note is the tight interactions formed with R106 (Figure 8C), a residue that is critical for the binding of classical NSAIDs bearing carboxylic acid moieties, such as indomethacin and flurbiprofen. In this regard, the binding mode of AA520 is very peculiar because it has, from one side, the key molecular interactions in common with selective COX-2 inhibitors, but still, some features recall the classical NSAIDs. For instance, NS-398 (Supplementary Figure S2A), one of the earliest COX-2 selective inhibitors, possesses a methanesulfonamide moiety interacting with the side chain of R120 (R106 according to the numbering employed herein), which has been indicated as a molecular determinant for time-dependent inhibition of COX-2 (Vecchio and Malkowski, 2011). NS-398 was initially expected to insert the methanesulfonamide moiety into the side pocket, similarly to the methylsulfone moiety of rofecoxib (Supplementary Figure S2B) however structural data proved that this group was, instead, positioned towards the constriction site. Compound AA520, thus, seems to recapitulate such behavior. In addition, the ability of AA520 to interact with R499, as observed for rofecoxib and other members of the coxib class, makes this ligand exquisitely selective. A good overlap between the phenyl ring, the lactone moiety of rofecoxib, and the *p*-chlorobenzoyl of AA520 within the central binding pocket could also be observed (Supplementary Figure S2B).

Lumiracoxib, reported to be the most potent COX-2-selective inhibitor *in vivo* (Blobaum and Marnett, 2007) lies within the central binding pocket. It has been found to interact with S516 and Y371 through its carboxylate moiety. These latter contacts have emerged from the MD simulation of AA520, highlighting the ability of this ligand to engage many critical interactions observed for potent and selective COX-2 inhibitors but also shared by classical NSAIDs.

The overlay of AA520 and indomethacin showed a certain overlap of their *p*-chlorobenzoyl groups (Supplementary Figures S2D, S3) as well as the 2′-methyl of indomethacin and the phenethyl linker of AA520. A hallmark of indomethacin inhibitory activity of COX enzymes is that it appears to be functionally irreversible; reversibility assays carried out by us in HCA7 cells confirmed this finding and displayed, for compound AA520, a behavior like indomethacin. Interestingly, the 2′-methyl group of indomethacin is projected in a pocket formed by V335, A513, S516, and L517 (Supplementary Figure S3). Mutations reducing the size of this pocket or removal of the 2′-methyl group convert indomethacin from a potent tight binding inhibitor to a rapidly reversible, weaker inhibitor (Prusakiewicz et al., 2004), suggesting that the interactions formed within this small and rather hydrophobic pocket may be involved in the formation of a tightly bound enzyme-inhibitor complex. Therefore, it is tempting to speculate that such a mechanism might also apply to AA520, being able to recapitulate such interaction patterns.

To sum up, AA520 selectivity and potency could mainly be ascribed to the benzenesulfonamide head group, which can engage both R106 and R499 (a key residue for COX-2 selectivity); the remainder of the ligand extends towards the central binding pocket, where it is stabilized by additional interactions, including the H-bonds with S516 and Y371, which are critical for binding of selective and potent inhibitors such as lumiracoxib.

### 3.8 Effects of AA520, GW6741 and rofecoxib on cell viability

We have previously demonstrated that AA520 exhibits antagonistic effects on PPARα using *in vitro* transactivation assay, with an IC_50_ of 0.80 ± 0.08 μM (mean ± SEM) (Ammazzalorso et al., 2016). Thus, we aimed to compare the effect of AA520 with a PPARα antagonist GW6471 (Xu et al., 2002) on the MTT cell viability/toxicity assay in HCA7 cancer cells. Moreover, we aimed to verify the contribution of COX-2 inhibition to this effect by coincubating GW6471 with rofecoxib.

**FIGURE 9:**
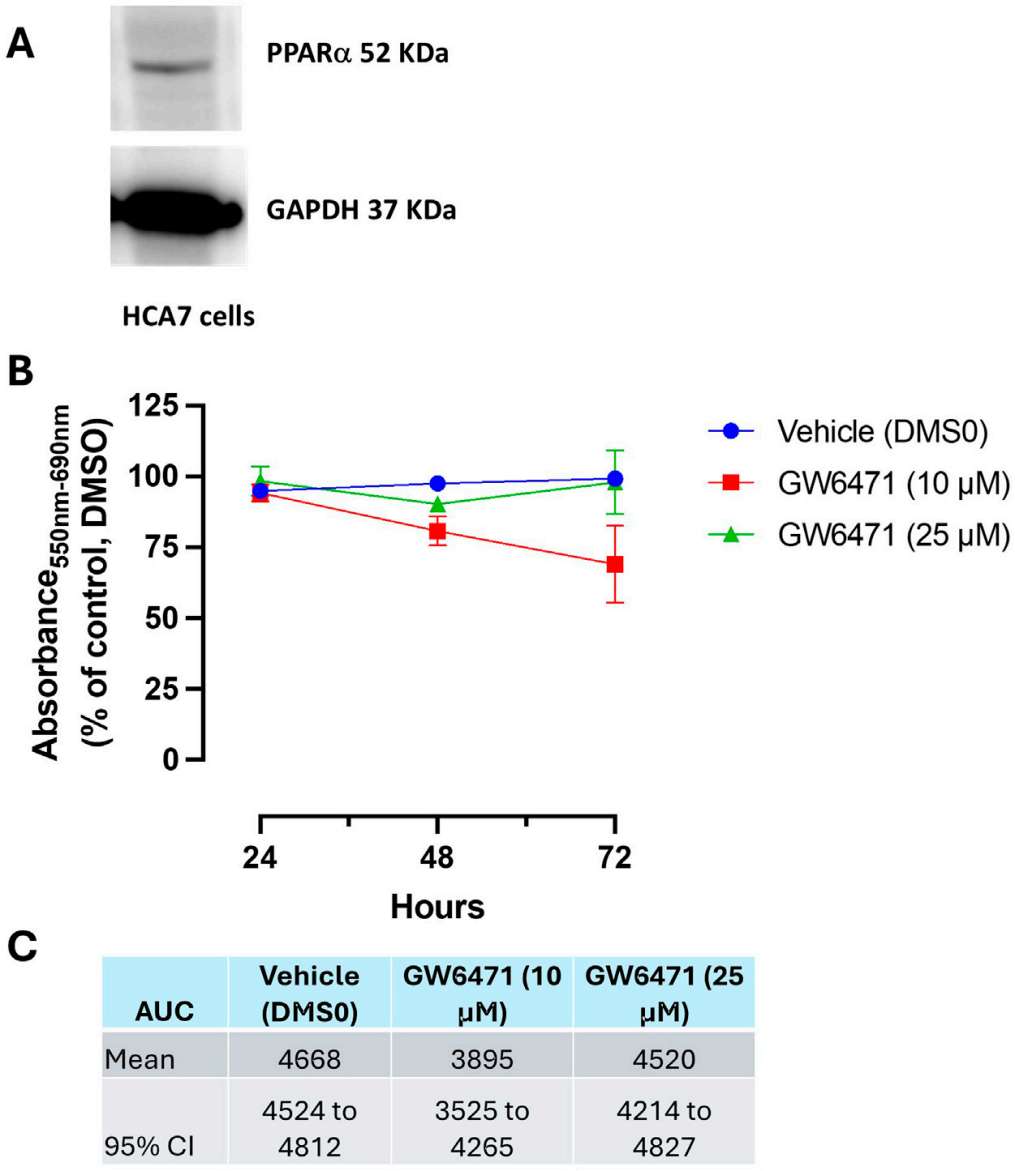
**(A)** Western blot analysis of PPARα in HCA7 cells. **(B, C)** Effect of GW6471 (a PPARα antagonist) on HCA7 cell viability. **(B)** GW6471 (10 and 25 μM) was added to HCA7 cells (4 × 10^3^ cells/well), and an MTT assay was performed for up to 72 h of incubation; results are expressed as a percent of control (DMSO) (mean ± SEM, n = 10). **(C)** AUC values were assessed from 24 to 72 h, providing the mean and 95% CI.

As shown in Figure 9A, HCA7 cells express PPARα, and we have previously shown that the cells also express COX-2 (Hofling et al., 2022; Tacconelli et al., 2020b) and generate PGE_2_. GW6471, at 10 μM, reduced MTT in a time-dependent fashion. A nonsignificant effect on MTT response was found at higher concentrations of the compound (Figures 9B, C).

**FIGURE 10:**
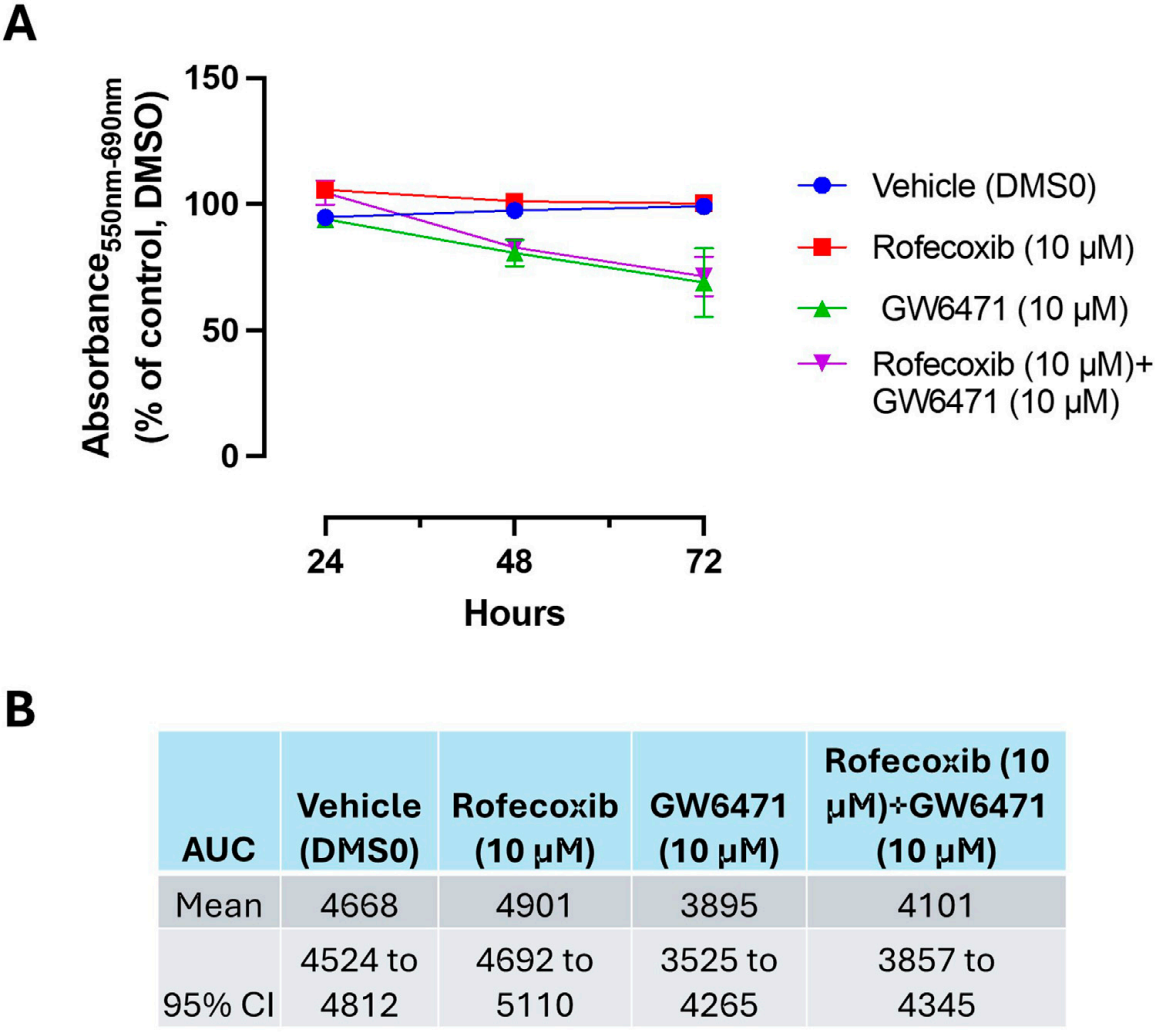
Effect of Rofecoxib and GW6471 on HCA7 cell viability. **(A)** Rofecoxib 10 μM, GW6471 10 μM, or both compounds were added to HCA7 cells (4 × 10^3^ cells/well), and an MTT viability assay was performed for up to 72 h of incubation; results are expressed as percent of control (DMSO) (mean ± SEM, n = 10). **(B)** AUC values were assessed from 24 to 72 h, providing the mean and 95% CI.

The MTT reduction by GW6471 (10 μM) was not influenced by the coincubation with rofecoxib. We used a concentration of 10 μM of rofecoxib, which caused a selective maximal inhibition of COX-2 activity (Figure 3). The selective COX-2 inhibitor incubated alone did not affect MTT (Figures 10A, B).

**FIGURE 11:**
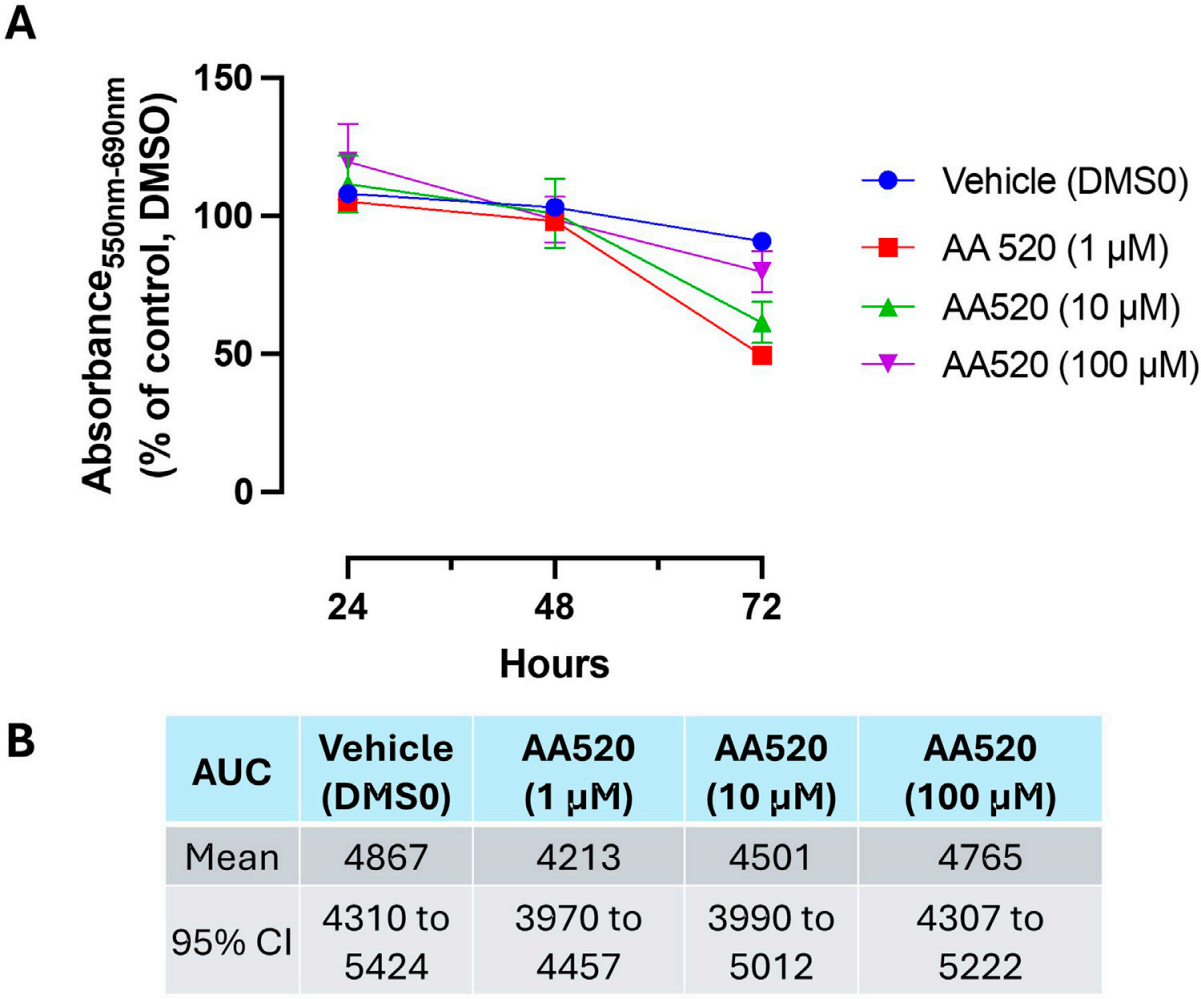
Effect of AA520 on HCA7 cell viability. **(A)** AA520 (1–100 μM) was added to HCA7 cells (4 × 10^3^ cells/well), and an MTT viability assay was performed for up to 72 h of incubation; results are expressed as a percent of control (DMSO) (mean ± SEM, n = 10). **(B)** AUC values were assessed from 24 to 72 h, providing the mean and 95% CI.

AA520 caused a maximal time-dependent MTT reduction at 1 μM, a concentration that inhibits PPARα and COX-2, at 72 h. This effect was reduced at higher concentrations (Figures 11A, B).

**FIGURE 12:**
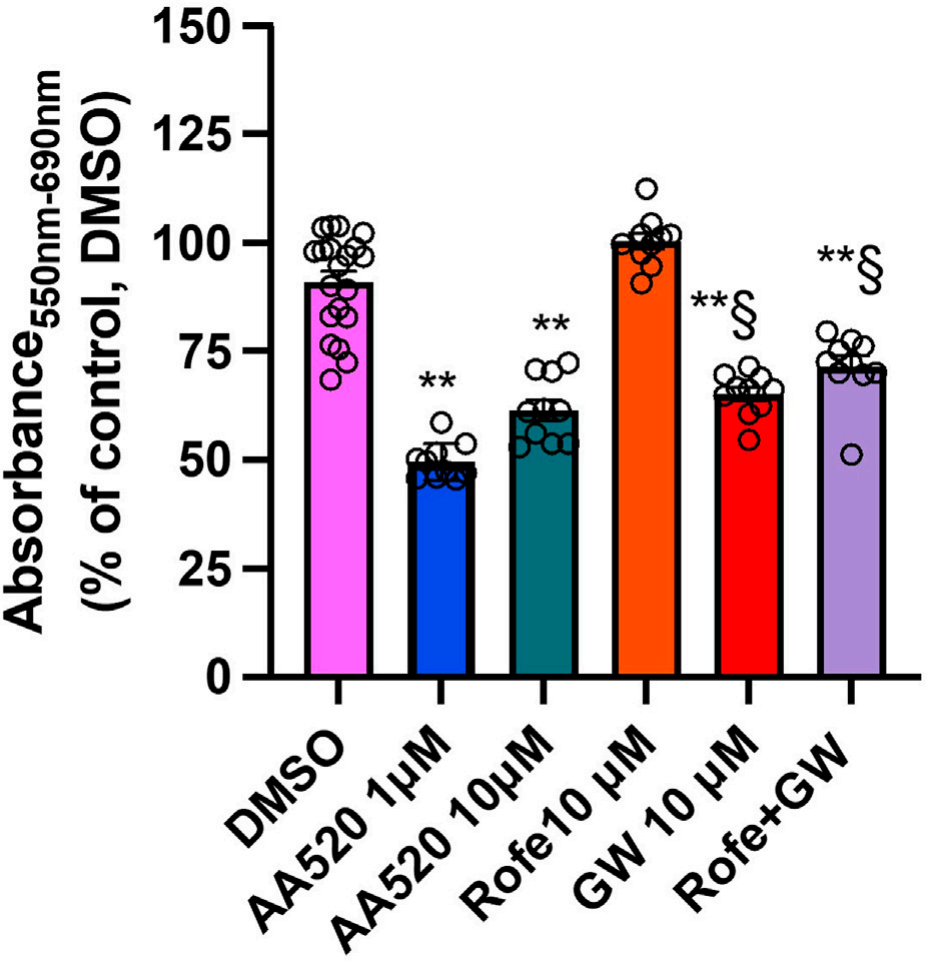
Comparison of the effects of AA520, rofecoxib, and GW6471 on HCA7 cell viability at 72 h. AA520 (1 or 10 μM), rofecoxib (10 μM), GW6471 (10 μM), or both rofecoxib and GW6471 were added to HCA7 cells (4 × 10^3^ cells/well) for 72 h, and an MTT viability assay was performed; results are expressed as a percent of control (DMSO) (mean ± SEM, n = 10–20). The data were analyzed using a one-way ANOVA, followed by Dunnett’s or Tukey’s test; **P < 0.01 versus vehicle; §P< 0.01 vs. AA520 1 μM.

Figure 12 reports the data found at 72 h of incubation. AA520 at 1 μM caused a more profound reduction of MTT than GW6471 (10 μM). Rofecoxib did not potentiate the MTT effect of the PPARα antagonism by GW6471. These data suggest that the contribution of PPARα antagonism is involved in the cytotoxic effect of AA520 in HCA7 cancer cells.

## 4 Discussion

With an *in silico* approach, we identified a novel chemical scaffold that is highly selective and potent in inhibiting COX-2 activity in inflammatory and cancer cells. AA520 is a sulfonamide derivative of bezafibrate, and we have previously shown that it is also a potent antagonist of PPARα (Ammazzalorso et al., 2016). Thus, our compound is a unique molecule with dual inhibitory effects on COX-2 and PPARα at the same concentration range.

To characterize the pharmacological effects of AA520 on COX-isozymes, we have used human whole-blood assays (Patrignani et al., 1994; Tacconelli et al., 2020a). We also evaluated whether the compound inhibits other enzymatic and nonenzymatic pathways involved in AA metabolism (Mazaleuskaya et al., 2018). To this aim, we assessed the main prostanoids PGE_2_ and TXB_2_ and some of the HETEs in both the R and S configurations in LPS-stimulated whole blood using LC-MS/MS. Human whole blood associated with LC-MS/MS is appropriate for the characterization of the effect of drugs on bioactive eicosanoid lipidomics *in vitro* and *ex vivo*, and it is ideal for drug screening (Mazaleuskaya et al., 2018). It allows small sample sizes and reproducible measures of a broad spectrum of eicosanoids in human blood. This assay can capture drug-induced substrate rediversion and unexpected shifts in product formation by blocking microsomal prostaglandin E synthase-1 (mPGES-1) inhibitors (Cheng et al., 2006). It can identify drug off-target effects. It can detect an antioxidant effect by assessing the levels of HETEs generated from AA by auto-oxidation (Powell and Rokach, 2015).

AA520 resulted in a highly selective and potent inhibitory effect on leukocyte COX-2 activity. The compound was >697-fold more potent towards leukocyte COX-2 than platelet COX-1. The AA520s highly selective inhibitory effect on COX-2 is due to its bulky molecular structure, which makes it difficult to bind the narrow active site of COX-1. Our study examined how AA520 interacts with COX-2 in the human colon cancer cell line HCA7, which does not express COX-1 (Hofling et al., 2022; Tacconelli et al., 2020b). We found that the compound strongly binds to the active site of COX-2, and this binding persists even after extensive washing. This type of binding is like the slow, time-dependent inhibition kinetics seen with COX inhibitors like indomethacin (Blobaum and Marnett, 2007). The docking and molecular dynamics experiments further supported the results from our biochemical characterization studies. The main determinants of AA520 selectivity and potency reside in the benzenesulfonamide head group, which can interact with R106 and R499 (a key residue for COX-2 selectivity). In addition, the hydrophobic contacts with V335, A513, S516, and L517 (Supplementary Figure S3) are likely to form a tightly bound enzyme-inhibitor complex, recapitulating the indomethacin interaction pattern. Interestingly, the benzenesulfonamide head group of AA520 is also a key structural requirement for the antagonistic activity of PPARα, as shown by previous molecular modeling studies (Ammazzalorso et al., 2016). AA520, thus, might be able to induce a receptor’s conformation, which is prone to co-repressor recruitment. While derivatives bearing benzothiazole (Ammazzalorso et al., 2016) or benzoxazole (Moreno-Rodríguez et al., 2024) rings present a dual α/γ inhibitory profile, AA520 is selective for PPARα.

As AA520 acts with a dual action mechanism, the antagonism of PPARα and the inhibition of COX-2, it may present potential immunomodulating and antineoplastic activities (Wagner and Wagner, 2022; Wang and Dubois, 2010). The antitumor effects of COX-2 inhibition are well documented since PGE_2_ is involved in proliferation, migration, and immune escape (Patrignani and Patrono, 2015). A PPARα antagonism action can improve the anticancer effect of COX-2 inhibition. PPARα transcription factor regulates fatty acid oxidation and inflammation in many cancers (Varga et al., 2011). TPST-1120, an orally bioavailable, small molecule, selective, and competitive antagonist of PPARα, is in clinical development by Tempests Therapeutics (Stock 2017). TPST-1120 has shown promise in killing tumor cells and promoting tumor-specific immunity (Whiting et al., 2019). In an ongoing Phase Ib/II, open-label, multicenter, randomized umbrella study in participants with advanced liver cancers, positive results were obtained in combination with atezolizumab (an immune checkpoint inhibitor) and bevacizumab (an antiangiogenic drug) (https://clinicaltrials.gov/study/NCT04524871).

We have tested the impact of AA520 on the MTT assay, which evaluates cell metabolism by estimating mitochondrial NAD(P) H oxidoreductases or cytoplasmic esterase activities (Braissant et al., 2020). This assay assesses the reduction in the number of viable cells. However, we did not study whether the reduction of MTT was due to inhibition of cell metabolism and/or proliferation (cytostatic effect) or actual cell death (cytotoxic effect). Further studies should clarify this issue using different cancer cell lines.

AA520 at 1 μM at 72 h caused an approximately 50% reduction of the MTT response in HCA7 cells, which was significantly higher than that of PPARα antagonist GW6471 (10 μM) (Xu et al., 2002). Both AA520 and GW6471 decreased the effect on MTT when used at higher concentrations. Several explanations can be suggested, such as the antagonists’ loss of specificity towards PPARα at higher concentrations. However, AA520 effectively reduced cellular metabolic activity as an indicator of cell viability, proliferation, and cytotoxicity at the appropriate low concentration, affecting PPARα and COX-2 activity.

AA520 can reduce inflammation and pain associated with tumors that exhibit high expression of both PPARα and COX-2, such as advanced RCC (Chen et al., 2004; Abu Aboud et al., 2013), for which there are no effective therapies that prevent its progression. The compound can help alleviate pain linked to tumor metastases, whether used alone or in combination with antiangiogenic and immune checkpoint inhibitors (Song et al., 2020).

AA520 demonstrates high selectivity in inhibiting COX-2, leading to a gastrointestinal safety profile. However, further investigation is needed to understand the impact of the dual inhibitory activity against COX-2 and PPARα on the cardiovascular system. Ongoing studies aim to characterize its effect on the biosynthesis of vascular prostacyclin in experimental models.

In conclusion, considering the synergistic effect between PPARα and COX-2 inhibitors in limiting tumorigenesis, the development of molecules with a dual pharmacological target, i.e., COX-2 inhibitors and PPARα antagonists, is of clinical relevance. This strategy can provide several advantages over single-target inhibitors (Löscher, 2021): it can reduce the risk of drug resistance, achieve greater anti-tumor efficacy, and minimize adverse events by possibly requiring lower drug dosing during treatment.

## Data Availability

The raw data supporting the conclusions of this article will be made available by the authors, without undue reservation.
