# Supplementary material for: A sulfonimide derivative of bezafibrate as a dual inhibitor of cyclooxygenase-2 and PPARα
Source: Front Pharmacol. 2024 Nov 26;15:1488722. doi: 10.3389/fphar.2024.1488722 (PMC11628281; doi:10.3389/fphar.2024.1488722)

Supplementary Material

Table S1. Cluster analysis of the MD simulation.

Cluster number	Cluster size
1	14
2	13
3	9
4	8
5	8
6	8
7	7
8	7
9	6
10	6
11	5
12	3
13	3
14	2
15	1

Table S2

ID	Structure	Docking Score[a]	PPARα IC_{50} (μM)[b]
**1d**	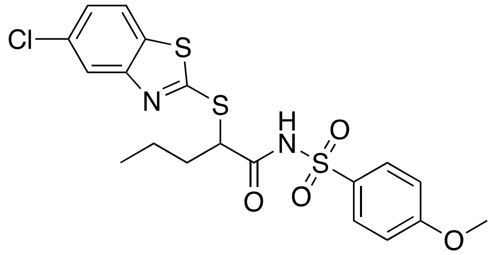	-7.816	0.68 ± 0.04
**5**	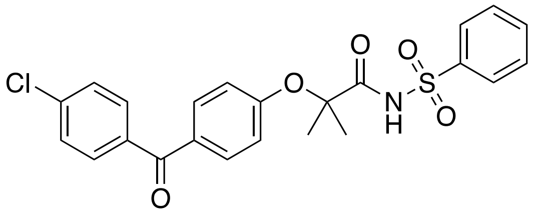	-7.204	7.65 ± 0.23
**2b**	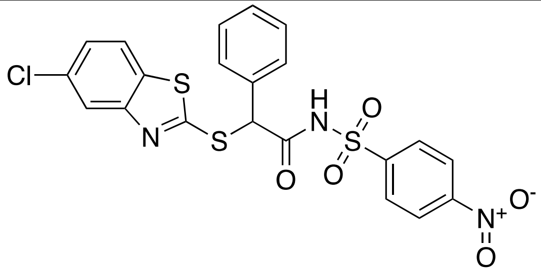	-6.920	0.82 ± 0.07

**7**	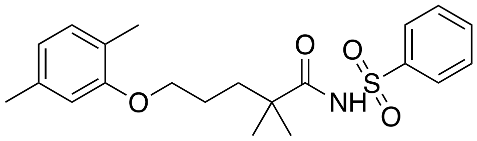	-6.011	8.80 ± 0.29
**6 (AA520)**	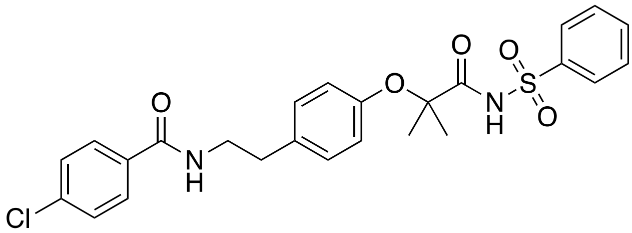	-5.738	0.80 ± 0.08
**1a**	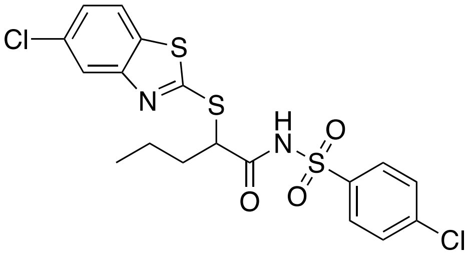	-5.256	6.86 ± 0.12
**4**	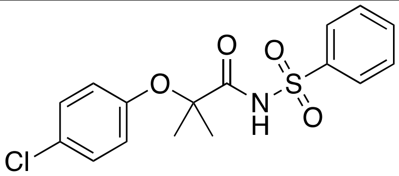	-5.172	0.65 ± 0.01
**1c**	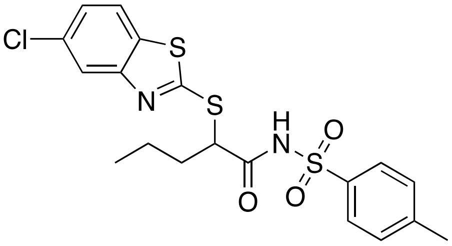	-5.046	4.60 ± 0.23
**1f**	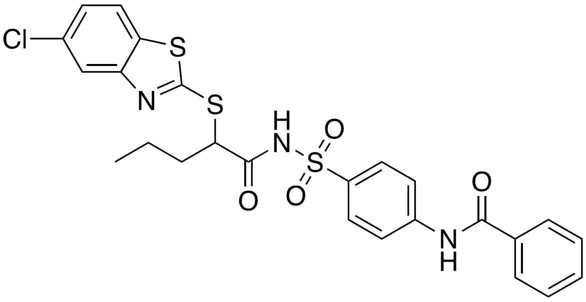	-4.152	4.50 ± 0.03

[a]Docking score refers to the virtual screening against COX-2, as detailed in 3.1 Section.
[b]The PPARα IC_{50} values are taken from Ammazzalorso et al., 2016.

Figure S1. 2D ligand interaction diagram of compounds AA520 into COX-2 (PDB 3LN1) as predicted by IFD calculations. Positively charged amino acids are represented with dark blue drops, negatively charged amino acids are represented with red drops, polar amino acids are represented with light blue drops and hydrophobic amino acids are represented with green drops. H-bonds are depicted with purple arrows. Green lines represent p-stacking interactions. Blue-red lines represent salt bridges.

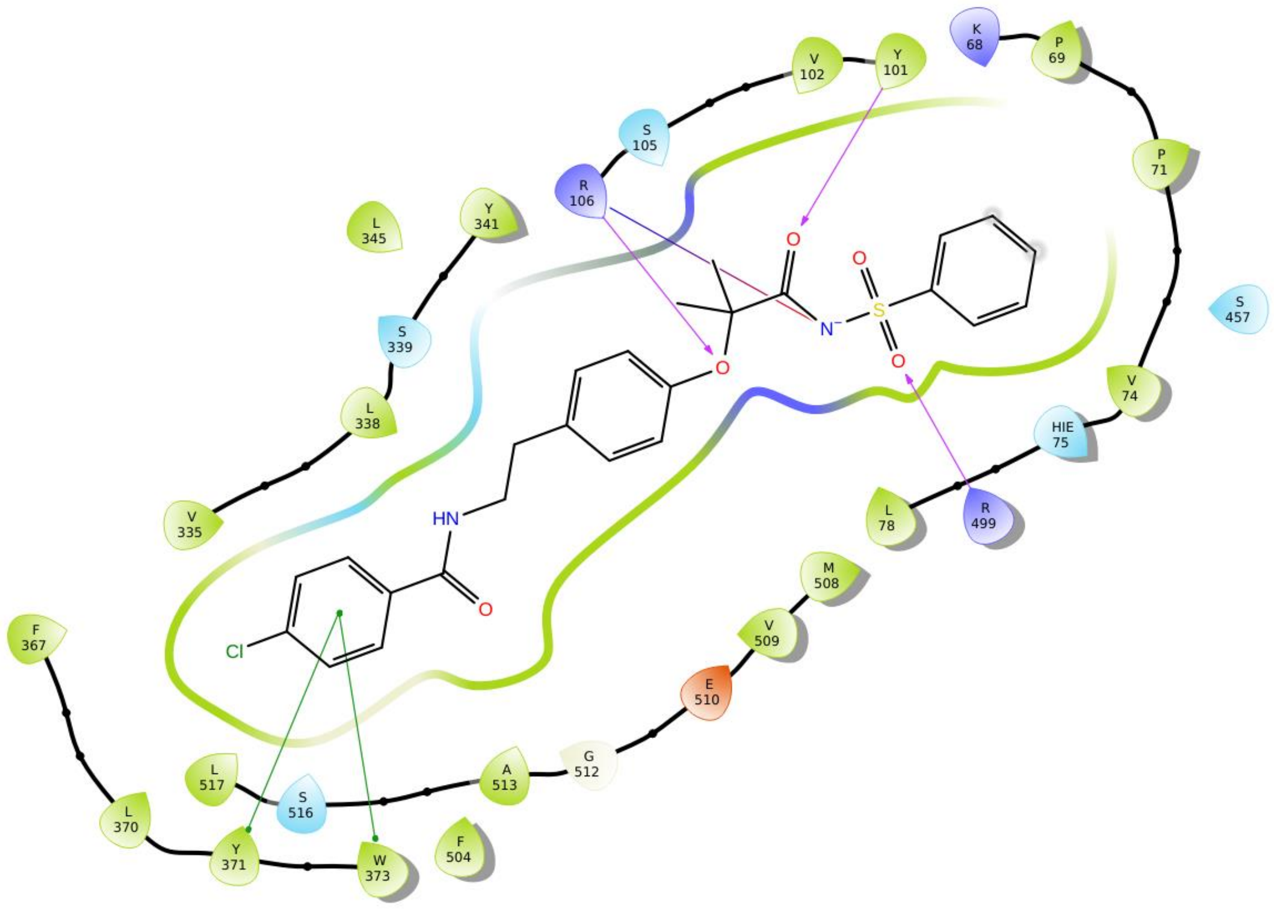

Table S3. Biosynthesis of eicosanoids in LPS-stimulated human whole blood in vitro.

	**Saline (NaCl 0.9%)**	**LPS (10μg/ml)**
**Eicosanoid, ng/ml**		
12R-HETE	0.663±0.22	0.90±0.22
12S-HETE	325.1±124.9	343.3±101.6
15R-HETE	3.125±0.60	10.33±1.71**
15S-HETE	7.40±2.88	10.98±3.31*
5R-HETE	2.54±0.69	8.18±1.034**
5S-HETE	1.55±0.24	2.36±0.70**
8R-HETE	1.27±0.45	1.34±0.35
8S-HETE	1.89±0.64	1.98±0.53
LTB_4	0.150±0.07	1.20±0.77**
15R-LXA_4	<0.01	<0.01
PGE_2	0.98±0.21	11.89±2.1**
TXB_2	2.99±0.69	13.68±0.57**

Aliquots of 1 ml of heparinized whole blood were incubated for 24 h at 37°C with physiological solution (0.9% NaCl, saline) or with LPS (10 μg/ml, dissolved in 0.9% NaCl). At the end of the incubation, the blood was centrifuged, and the levels of the different eicosanoids were measured. The data of the different eicosanoids are reported as mean ± SD.

* $P<0.05$, ** $P<0.01$ versus Saline (NaCl 0.9%) (n=8)

Figure S2. Overlay of AA520 (violet sticks) bound to COX-2 (PDB 3LN1) after 100 ns MD (the representative structure from the most populated cluster is shown) with (A) NS-398 (yellow sticks, PDB 3QMO); (B) rofecoxib (gray sticks, PDB 5KIR); (C) lumiracoxib (slate sticks, PDB 4OTY); (D) indomethacin (dark salmon sticks, PDB 4COX)

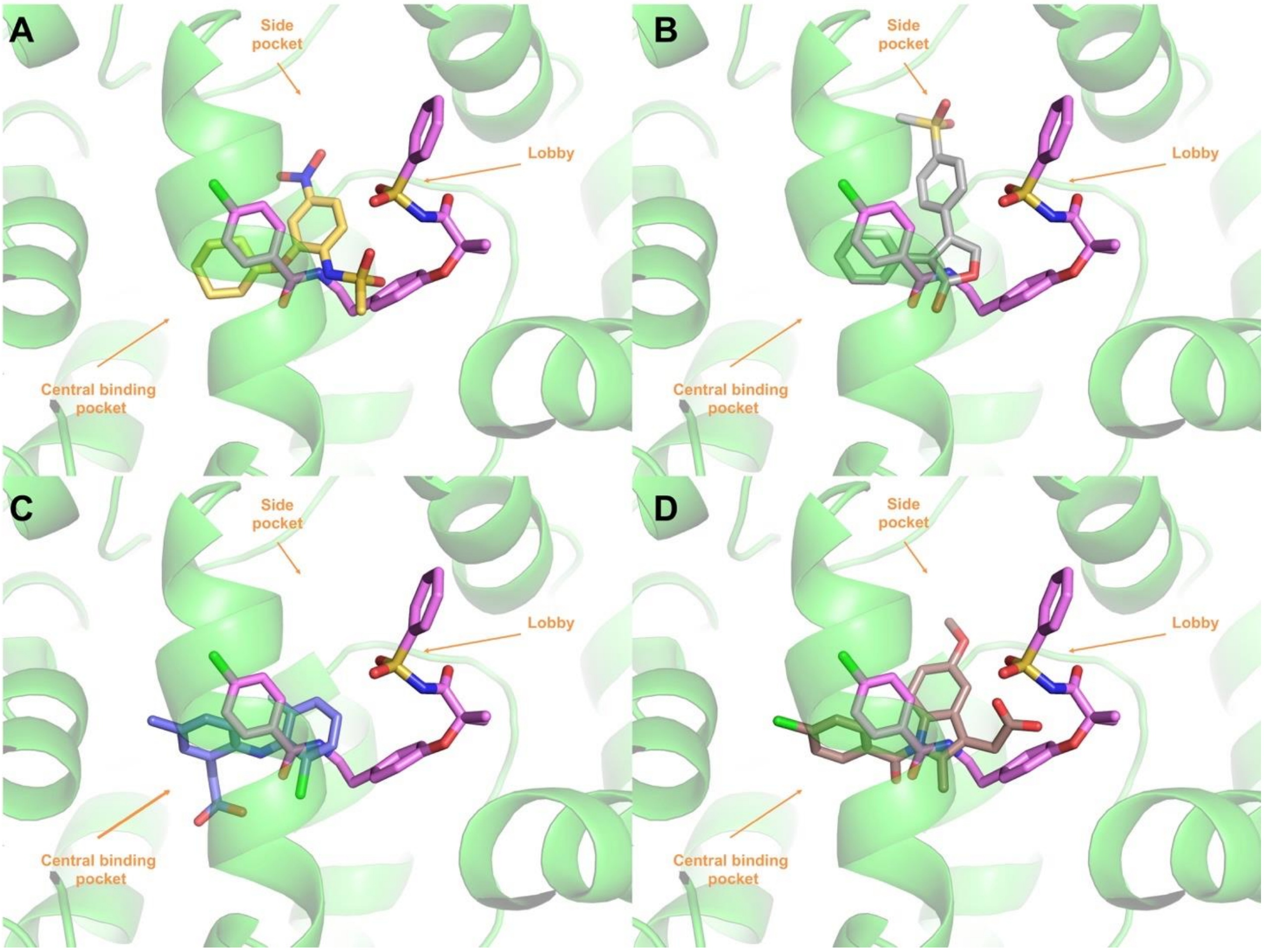

Figure S3. Zoom-in of the overlay of AA520 (violet sticks) bound to COX-2 (PDB 3LN1) after 100 ns MD (the representative structure from the most populated cluster is shown) with indomethacin (dark salmon sticks, PDB 4COX). The amino acid residues lining the pocket (V335, A513, S516, and L517), in which the 2’-methyl group of indomethacin is projected, are displayed as white sticks and labeled.

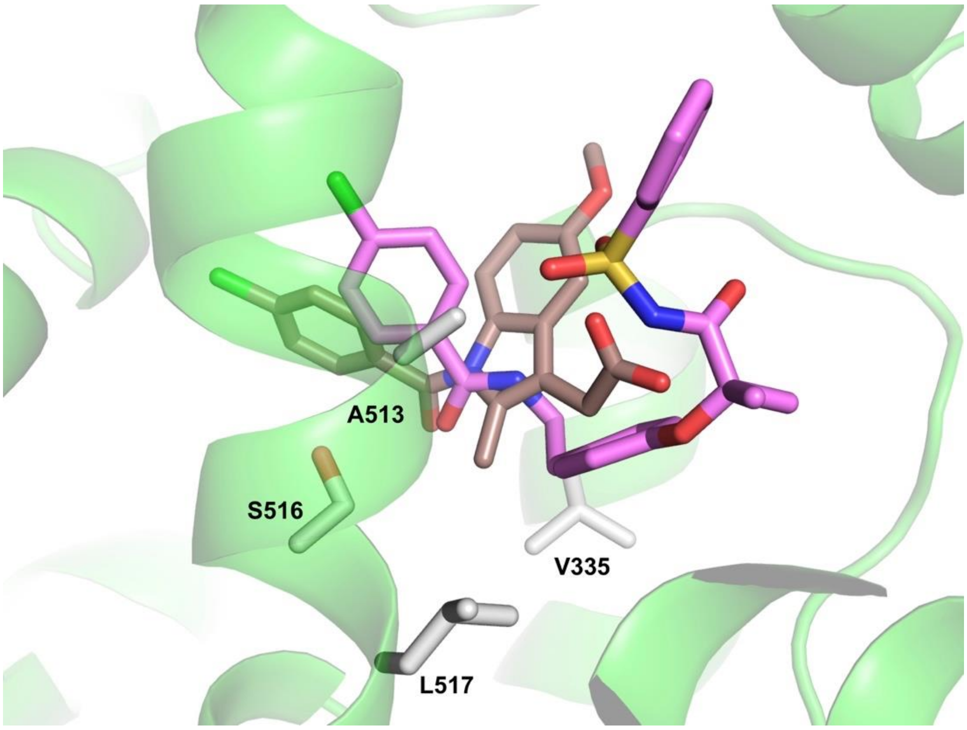

Figure S4. Uncropped gel of western blotting membrane presented in Figure 4A

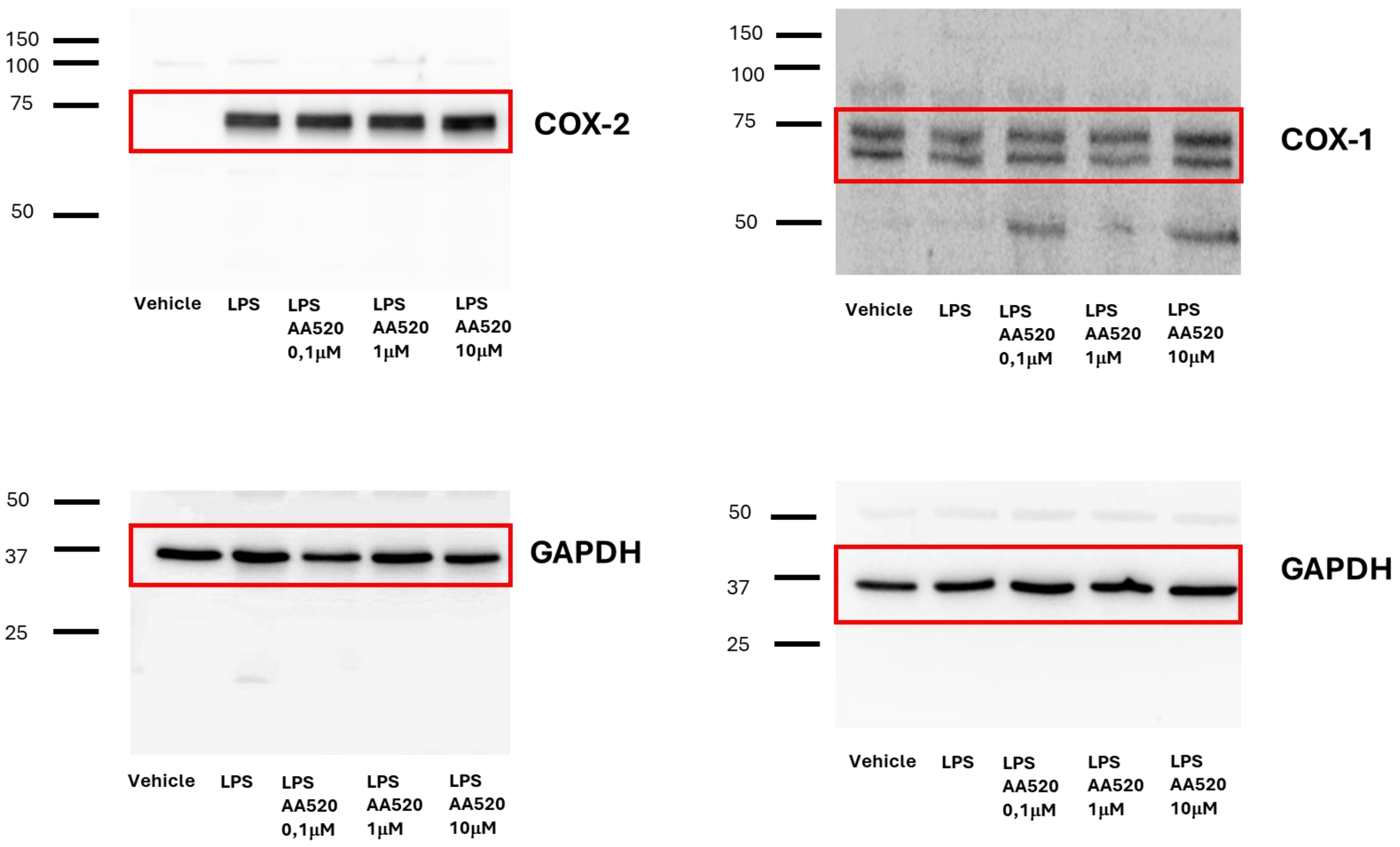

Figure S5: Uncropped gel of western blotting membrane presented in Figure 9A

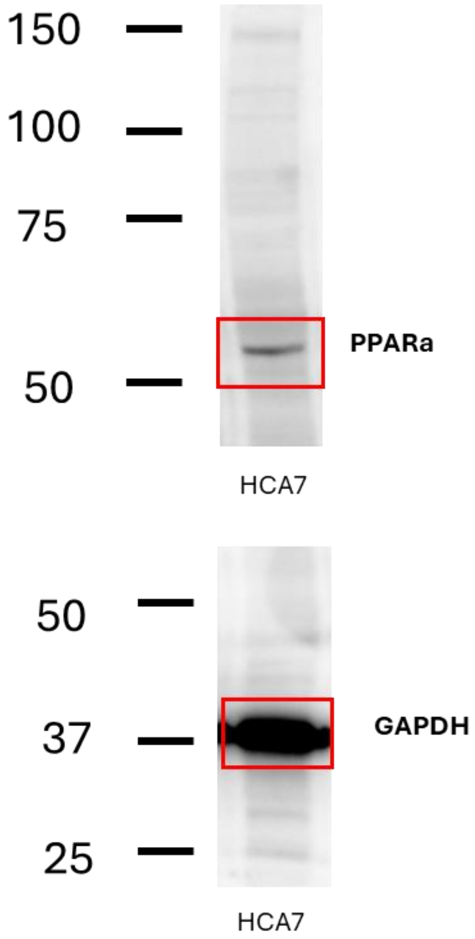

Supplement: Supplementary file 1 [file DataSheet1.PDF]
